# Comprehensive genome-wide analysis of the pear (*Pyrus bretschneideri*) laccase gene (*PbLAC*) family and functional identification of *PbLAC1* involved in lignin biosynthesis

**DOI:** 10.1371/journal.pone.0210892

**Published:** 2019-02-12

**Authors:** Xi Cheng, Guohui Li, Chenhui Ma, Muhammad Abdullah, Jinyun Zhang, Hai Zhao, Qing Jin, Yongping Cai, Yi Lin

**Affiliations:** 1 School of Life Science, Anhui Agricultural University, Hefei, China; 2 Horticultural Institute, Anhui Academy of Agricultural Sciences, Hefei, Anhui, China; Indiana University, UNITED STATES

## Abstract

The content and size of stone cell clusters affects the quality of pear fruit, and monolignol polymerization and deposition in the cell walls constitute a required step for stone cell formation. Laccase (LAC) is the key enzyme responsible for the polymerization of monolignols. However, there are no reports on the *LAC* family in pear (*Pyrus bretschneideri*), and the identity of the members responsible for lignin synthesis has not been clarified. Here, 41 *LAC*s were identified in the whole genome of pear. All *Pyrus bretschneideri LAC*s (*PbLAC*s) were distributed on 13 chromosomes and divided into four phylogenetic groups (I-IV). In addition, 16 segmental duplication events were found, implying that segmental duplication was a primary reason for the expansion of the *PbLAC* family. *LAC*s from the genomes of three Rosaceae species (*Prunus mummer*, *Prunus persica*, and *Fragaria vesca*) were also identified, and an interspecies collinearity analysis was performed. The phylogenetic analysis, sequence alignments and spatiotemporal expression pattern analysis suggested that *PbLAC1*, *5*, *6*, *29*, *36* and *38* were likely associated with lignin synthesis and stone cell formation in fruit. The two target genes of *Pyr-miR1890* (a microRNA identified from pear fruit that is associated with lignin and stone cell accumulation), *PbLAC1* and *PbLAC14*, were selected for genetic transformation. Interfamily transfer of *PbLAC1* into Arabidopsis resulted in a significant increase (approximately 17%) in the lignin content and thicker cell walls in interfascicular fibre and xylem cells, which demonstrated that *PbLAC1* is involved in lignin biosynthesis and cell wall development. However, the lignin content and cell wall thickness were not changed significantly in the *PbLAC14*-overexpressing transgenic Arabidopsis plants. This study revealed the function of *PbLAC1* in lignin synthesis and provides important insights into the characteristics and evolution of the *PbLAC* family.

## Introduction

*Pyrus bretschneideri* cv. ‘Dangshan Su’ is one of the most important exported fruits in China and is well known throughout the world for its rich nutritional and medicinal value, but one of the disadvantages of this variety is the large diameter of the stone cell clusters (SCCs) and their high abundance in the fruit [1,2]. The content and diameter of SCCs in pear fruit are negatively correlated with the content of sucrose and cause a gritty texture and coarse mouthfeel. An excessive abundance and/or an increased diameter of SCCs affect the fruit flavour and consumer appreciation [3–5]. Therefore, the content and diameter of SCCs are key factors affecting the quality of pear fruit.

The SCCs in pear fruit are composed of multiple stone cells. It has been clarified that stone cells are a type of solid lignification cells and differentiated from the parenchyma cells of the flesh [6,7]. An analysis of the cell wall composition of stone cells in mature pear fruit showed that each gram of cell wall material contains 156 mg of lignin, whereas the parenchyma cell walls contain only 17 mg of lignin per gram of cell wall material [8]. In addition, a large amount of lignin is deposited in the compound middle lamella (CML) and in each layer of the secondary cell wall (SCW) of stone cells [1,6], which indicates that lignin is one of the main components of stone cells and that the biosynthesis of lignin is closely related to stone cell formation [9–11]. Therefore, controlling the synthesis and deposition of lignin in pear fruit would constitute a major strategy for inhibiting stone cell formation and thereby increasing the quality of pear fruit [12,13]. In recent years, many studies on structural genes related to lignin biosynthesis have been published, but only a few of these studies focused on genes related to lignin monomers polymerization, such as the gene encoding laccase.

Laccase (EC1.10.3.2) is the largest subfamily of multi-copper oxidases (MCOs) and has a wide range of substrates, including oxidized bisphenol, amino- or methoxy-substituted monophenols, aromatic diamines, hydroxylamine ammonia components and monolignols [14–16]. In plants, laccase is primarily involved in monomer polymerization to form phenolic biopolymers and in the stress response [17–19].

The function of the laccase family in the metabolism of lignin has been studied in model organisms and economically important species, such as *Arabidopsis thaliana*, *Brachypodium distachyon*, *Gossypium arboreum*, and *Oryza sativa* [17,18,20,21]. Among the 17 *LAC*s that have been identified in Arabidopsis, previous studies have investigated three *LAC*s (*AtLAC4*, *11* and *17*) that are responsible for lignin polymerization [22], and the results showed that the lignin content was slightly decreased in the double mutants *Atlac4 lac11* and *Atlac4 lac17* and substantially decreased in the triple mutant *Atlac4 lac11 lac17*, indicating a functional redundancy among these *LAC*s [20,22]. Additionally, published articles have reported that *AtLAC15*/*TRANSPARENT TESTA* 10 has dual functions, as indicated by its ability to simultaneously catalyse the polymerization of flavonoids and monolignols [16,19]. The study of 29 laccase family members in *B*. *distachyon* revealed that only *BdLAC5* is responsible for the polymerization of lignin [17]. Liu et al. (2017) screened 30 *LAC*s in *O*. *sativa* and found that, surprisingly, *OsLAC10* was not only associated with lignin synthesis but also involved in the abiotic stress response. Thus, *LAC* exists as a gene family in the plant genome, and many members have multiple overlapping functions. However, there is currently no systematic understanding of the pear laccase gene family, and it remains unclear which members play a role in the metabolism of fruit lignin.

Some recently published studies confirmed that laccase is regulated by microRNAs and affects plant lignin metabolism [23,24]. We also identified a differentially expressed microRNA (*Pyr-miR1890*) from two pear fruits with different stone cell and lignin contents [13]. The target genes of *Pyr-miR1890* are also *PbLAC*s (Pbr003857.1 and Pbr018935.1), and their expression levels exhibit opposite tendencies. Therefore, *Pyr-miR1890* might regulate the expression of these two *PbLAC*s and thereby affect lignin metabolism to change the stone cell content in pear fruit [13]. However, the function of Pbr003857.1 and Pbr018935.1 in lignin synthesis has not been further verified.

To clarify the role of the laccase gene family in lignin metabolism and stone cell development in pear fruit, we performed the first bioinformatics analysis aiming to identify and analyse the members of the *PbLAC* family, and the study included analyses of sequence properties, gene structures, conserved motifs, chromosome distribution, *cis*-acting elements, gene duplication, evolutionary relationship, and spatiotemporal expression patterns. The two target genes of *Pyr-miR1890* (Pbr003857.1 and Pbr018935.1, named *PbLAC1* and *PbLAC14*, respectively) were cloned and transformed into wild-type Arabidopsis (WT) to analyse their function in lignin metabolism. Thus, this study not only provides further insights into the characteristics and evolutionary relationship of the *PbLAC* family but also lays the foundation for the regulation of lignin synthesis and stone cell development in pear.

## Materials and methods

### Identification and sequence analysis of *LAC* family members in pear

Genomic data for pear were downloaded from GigaDB (http://gigadb.org/dataset/10008) [12]. The local protein database was constructed using BioEdit (http://www.mbio.ncsu.edu/bioedit/bioedit.html), and the conserved plant laccase domains (Cu-oxidase_2: PF07731, Cu-oxidase_3: PF07732, and Cu-oxidase: PF00394) were obtained from Pfam (http://pfam.xfam.org/). The three conserved laccase domains were then used as the query sequence for a Blastp search (E = 0.001) of the local protein database. The candidate sequences no conserved laccase domain were deleted. Gene family and protein domain identification was performed used the Pfam and SMART databases (http://smart.embl-heidelberg.de/), and all the members of the *PbLAC* family were obtained. Some basic information about the PbLACs was predicted, and the isoelectric point (pI) and molecular weight (MW) were predicted using ProtParam3 (http://web.expasy.org/protparam/). SignalP 4.1 (http://www.cbs.dtu.dk/services/SignalP/) was used to predict the signal peptide, and potential glycosylation sites were analysed using the NetNGlyc 1.0 online programme (http://www.cbs.dtu.dk/services/NetNGlyc/). The three-dimensional structures of the PbLACs were predicted using the Protein Fold Recognition server (www.sbg.bio.ic.ac.uk/~phyre2/html/page.cgi?id=index).

### Phylogenetic classification, gene structures and conserved motifs of *LAC* family members

The amino acid sequences of all laccase genes were used to construct phylogenetic trees, and sequence alignment was performed using the ClustalW function in MEGA 5.1 [25]. N-J phylogenetic trees were built with MEGA 5.1, and bootstrap analysis was conducted using 1000 replicates. The amino acid sequence information used to construct the phylogenetic tree is provided in S1 Table.

The conserved motifs of PbLACs were searched with MEME (http://meme-suite.org/tools/meme) [26]. Specifically, we searched for 20 conserved motifs, and the default values were used for the other parameters. The exon-intron structures were analysed using the Gene Structure Display Server (http://gsds.cbi.pku.edu.cn/) [27].

### Analysis of the *cis*-elements and chromosomal locations of the *PbLAC*s

We found the 2000-bp sequence upstream from each *PbLAC* initiation codon and used the online tool PlantCARE (http://bioinformatics.psb.ug/beto/webtools/plantcare/html.html) for the prediction of *cis*-elements.

We used MapInspect software to draw an image showing the chromosomal locations of the *PbLAC*s [28]. Genomic data for *Prunus mummer* (mei) were downloaded from GigaDB (http://gigadb.org/dataset/10008), and genomic data for *Prunus persica* (peach) and *Fragaria vesca* (strawberry) were obtained from the Phytozome database (https://phytozome.jgi.doe.gov/pz/portal.html). A collinearity analysis was performed using the Plant Genome Duplication Database (PGDD) [29]. The ratio of the non-synonymous substitution rate (Ka) to the synonymous substitution rate (Ks) and the sliding window of the duplicate genes were obtained with DNA Sequence Polymorphism 5.0 [30].

### RNA isolation and quantitative real-time PCR (qRT-PCR)

We collected flowers, buds, stems, leaves and fruits from 50-year-old pear trees planted in Dangshan County, Anhui Province, China, and all the samples were stored at -80°C until use. Fruits were collected at a eight time points, namely, 23, 39, 47, 55, 63, 79, 110 days after flowering (DAF) and at maturity (145 DAF).

Total RNA from each sample was isolated using a total RNAprep Pure Plant Kit (Tiangen, China). Reverse transcription was performed using a PrimeScript 1st Strand cDNA Synthesis Kit (TaKaRa, China) in accordance with the instructions provided with the kit. RNA purity (A_260_/A_280_ ratio) between 1.90 and 2.00 was used for subsequent experiments.

qRT-PCR was performed using TransStart Green qPCR SuperMix (TransGen Biotech, China) with a CFX96 Touch Real-Time PCR Detection System (Singapore). Each 20 μL reaction mixture consisted of 6.4 μL of nucleasefree water, 10.0 μL of SYBR Premix Ex Taq II, 0.8 μM of each primer, and 2 μL of diluted cDNA (500 ng/μL). Three biological replicates were performed for each sample. The relative expression level was calculated as 2^-ΔΔCt^ [ΔCt = Ct_Target gene_-Ct_Reference gene_. ΔΔCt = ΔCT_treatment_-ΔCT_control_] [31]. The qRT-PCR primer sequences are listed in S2 Table.

### Pear RNA-Seq data analysis

RNA-Seq data were downloaded from the Sequence Read Archive (SRA) (https://www.ncbi.nlm.nih.gov/sra) with the access numbers SRR5965146, SRR5965147, SRR5965142, SRR5965143, SRR5965144 and SRR5965145 [2]. The fragments per kilobase of exon per million fragments mapped (FPKM) values were obtained from the RNA-Seq data. The expression level of each *PbLAC* was displayed in the form of a heatmap using TBtools software (http://www.tbtools.com/).

### Overexpression of *PbLAC1* and *PbLAC14* in *Arabidopsis thaliana*

Based on the sequence information obtained from the genome, specific primers (S2 Table) were designed to amplify the *PbLAC1* and *PbLAC14* CDS, and two restriction sites (*Nco* I and *Bgl* II) were introduced at the ends (one in each end). The double restriction fragments were connected with the expression vector pCAMBIA1304 (GenBank: AF234300.1) using T_4_ ligase and sequenced, and the recombinant plasmids were verified. The plant expression vectors pCAMBIA1304-*PbLAC1* and pCAMBIA1304-*PbLAC14* were obtained and introduced into *Agrobacterium tumefaciens* EHA105 by electroporation.

The genetic transformations of *Arabidopsis thaliana* were accomplished using the floral dip method [32]. The transgenic Arabidopsis plants were screened with hygromycin (Hyg) (50 mg/L) and identified by RT-PCR and *β*-glucuronidase (GUS) staining. DNA extraction from Arabidopsis was performed using the EasyPure Plant Genomic DNA Kit (TransGen Biotech, China). The Arabidopsis DNA was used as the template to amplify the green fluorescent protein (*gfp*) CDS and thus determine whether successful integration into the genome was achieved. The amplified primers are listed in S2 Table. GUS was examined using a GUS Histochemical Assay Kit (Real-Times, China) according to the manufacturer’s recommended protocol. The methods used for the extraction and reverse transcription of Arabidopsis RNA were the same as those used for pear RNA.

### Histochemical staining of Arabidopsis and determination of lignin content

The inflorescence stems of the T_3_ generation of transgenic Arabidopsis were hand-sectioned for 2 months. The sections at the bottom portion (approximately 4 cm) of the inflorescence stems were then placed on glass slides, staining using the Wiesner staining method or 1% toluidine blue, and directly observed with a microscope [17,33,34].

The lignin content of the inflorescence stem of Arabidopsis was determined using the acetyl bromide method, which was previously described by Anderson et al. (2015) [34].

### Transmission electron microscopy (TEM)

The TEM observations were performed using the method described by De et al. (2017) [35]. The cell wall thickness of the cells was measured using the TEM images as described previously [36]. The cell wall thickness measurement software was Image-pro plus 6.0 (Media Cybernetics, Inc., Rockville, MD, USA).

### Statistical analyses

The statistical analyses were performed using Statistical Program for Social Sciences (release 19.0, SPSS Inc, IBM, www.ibm.com) and Microsoft Excel 2010.

## Results

### Identification and characterization of *LAC*s in the pear genome

We identified the laccase family in the local protein database through a Blastp search using the conserved domain shared by plant laccases. After deletion of the redundant sequences, we searched for the conserved domain using the Pfam and SMART databases. Forty-one *PbLAC*s, named *PbLAC1*-*PbLAC41*, were identified in the whole genome of pear (S3 Table). The lengths of the 41 PbLACs range from 1136 amino acids (aa) (PbLAC18) to 485 aa (PbLAC2), and their MWs range from 125.22 kDa (PbLAC18) to 53.3 kDa (PbLAC2). In addition, most of the PbLACs have an alkaline pI. Almost 70% of the PbLACs contain a signal peptide, and all the signal sequences allow the extracellular secretion of the laccase proteins. Similar to most Arabidopsis and rice laccases, all PbLACs are N-glycosylated glycoproteins [18].

### Analyses of the evolutionary, exon-intron structure and motif distribution of *PbLAC* family members

As shown in S1A Fig, the 41 *PbLAC*s can be divided into six subfamilies (I-VI), and a total of 13 gene pairs were obtained: *PbLAC1/14*, *PbLAC3/30*, *PbLAC6/36*, *PbLAC7/8*, *PbLAC10/11*, *PbLAC13/28*, *PbLAC15/31*, *PbLAC17/18*, *PbLAC20/26*, *PbLAC21/40*, *PbLAC22/37*, *PbLAC23/38* and *PbLAC33/34*. All of these gene pairs, with the exception of *PbLAC10/11*, were supported by high bootstrap values (bootstrap values ≥ 99).

To further clarify the characteristics of the pear laccase family, the distributions and types of conserved motifs were detected based on the PbLAC evolutionary relationships. Using MEME, we found 20 conserved motifs among the 41 PbLACs (S1B Fig), and we then used NCBI (https://www.ncbi.nlm.nih.gov/cdd) and Pfam to annotate their functions (S4 Table).

Motifs 1–3 encode three cupredoxin domains that belong to typical plant laccases. Specifically, motifs 1 and 2 are located at the N- and C-terminal regions of PbLACs, and motif 3 is mainly distributed in the middle. An analysis of the laccase family in pear revealed that PbLAC10, 12, 27, and 34 apparently lack motif 2, PbLAC17 does not contain motif 3, and the remaining PbLACs contain motifs 1, 2 and 3, which indicated the reliability of the screening and identification results. In addition, motif 16 was not shared by the IV and V subfamily members, signifying that the members of these two subfamilies might have lost this motif during the evolutionary process, resulting in a new function.

To better understand the structural features of *PbLAC*s, we analysed their exon-intron structure (S1C Fig). According to the number of introns and exons, the *PbLAC*s can be grouped into six classes. The first class of *PbLAC*s contains 11 introns and 12 exons and includes only *PbLAC18*, and the second class has 10 introns and 11 exons and includes *PbLAC34*. The third class has six introns and seven exons and includes four members, namely, *PbLAC10*, *20*, *26*, and *29*. In addition, the fourth class has five introns and six exons and contains a total of 22 members; the fifth class has four introns and five exons and includes eight members; and the sixth class has three introns and four exons and contains *PbLAC2*, *3*, *12*, *30*, and *37* (S1C Fig). Overall, the family members with closer genetic relationships have more similar exon-intron structures and motif distributions, which further demonstrates the reliability of the phylogenetic tree.

### Analysis of the upstream regulatory sequences of the *cis*-elements of *PbLAC*s

**Fig 1:**
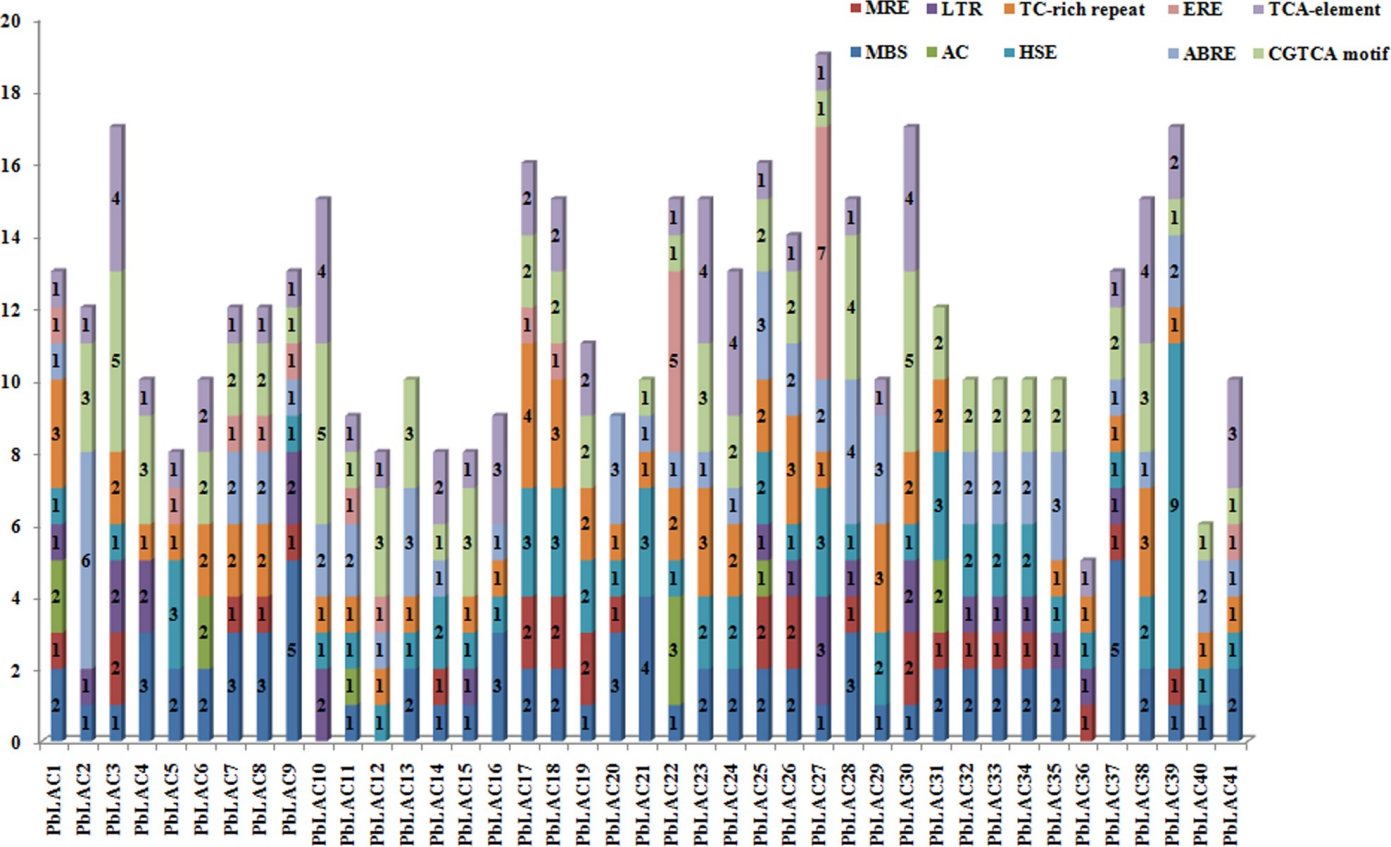
Analysis of *cis*-acting elements of *PbLAC*s. Different *cis*-elements are shown in different colours. The numbers represent the numbers of *cis*-elements in the regulatory sequence upstream of the gene.

To obtain further insights into the possible expression regulation mode of *PbLAC*s, we analysed the *cis*-acting elements of the 2000-bp regulatory sequence upstream of the 41 *PbLAC* coding sequences (CDS) (Fig 1, S5 Table).

After scanning the results, we found a large number of hormone- responsive *cis*-acting elements in the upstream regulatory sequences of *PbLAC* family members. Specifically, 30 members contain abscisic acid (ABA)-responsive elements (ABREs), 12 members have ethylene-responsive elements (EREs), 35 members contain the methyl jasmonate (MeJA)-responsive element (CGTCA motif), and 32 members contain the salicylic acid (SA)-responsive element (TCA element). These hormones are widely involved in the signalling pathways of mature senescence or the stress response [37,38], which suggests that *PbLAC* family members are likely to participate in ripening and the stress response in pear.

Furthermore, we identified some biotic and abiotic stress-related *cis*-acting elements in the upstream regulatory sequences of the *PbLAC*s, such as the TC-rich repeat element (related to defence) and the high-temperature stress-related (HSE), low-temperature stress-related (LTR), and drought stress-related (MBS) elements (S5 Table). These results suggest that members of the *PbLAC* family might play roles in the responses to a variety of abiotic and biotic stresses. Interestingly, 38 and 21 *PbLAC*s have MBS elements and MRE elements (involved in responses to light), respectively, which indicates that the expression of these members is regulated by drought stress or light.

### Chromosome location and gene duplication events of *PbLAC*s

To explore the chromosomal distribution and gene expansion factors of the *PbLAC* family, the chromosome localization and gene duplication of *PbLAC*s were analysed using MapInspect and DnaSP software, respectively. As shown in S2 Fig, the *PbLAC* family members are unequally distributed among 13 chromosomes in pear. Among these chromosomes, Chr6, Chr10 and Chr14 each contain only one member of the *PbLAC* family, and Chr11 contains the largest number of members, with a total of 6. Moreover, all the members of the *PbLAC* family on Chr1, Chr4, and Chr11 exist in the form of gene clusters.

**Fig 2:**
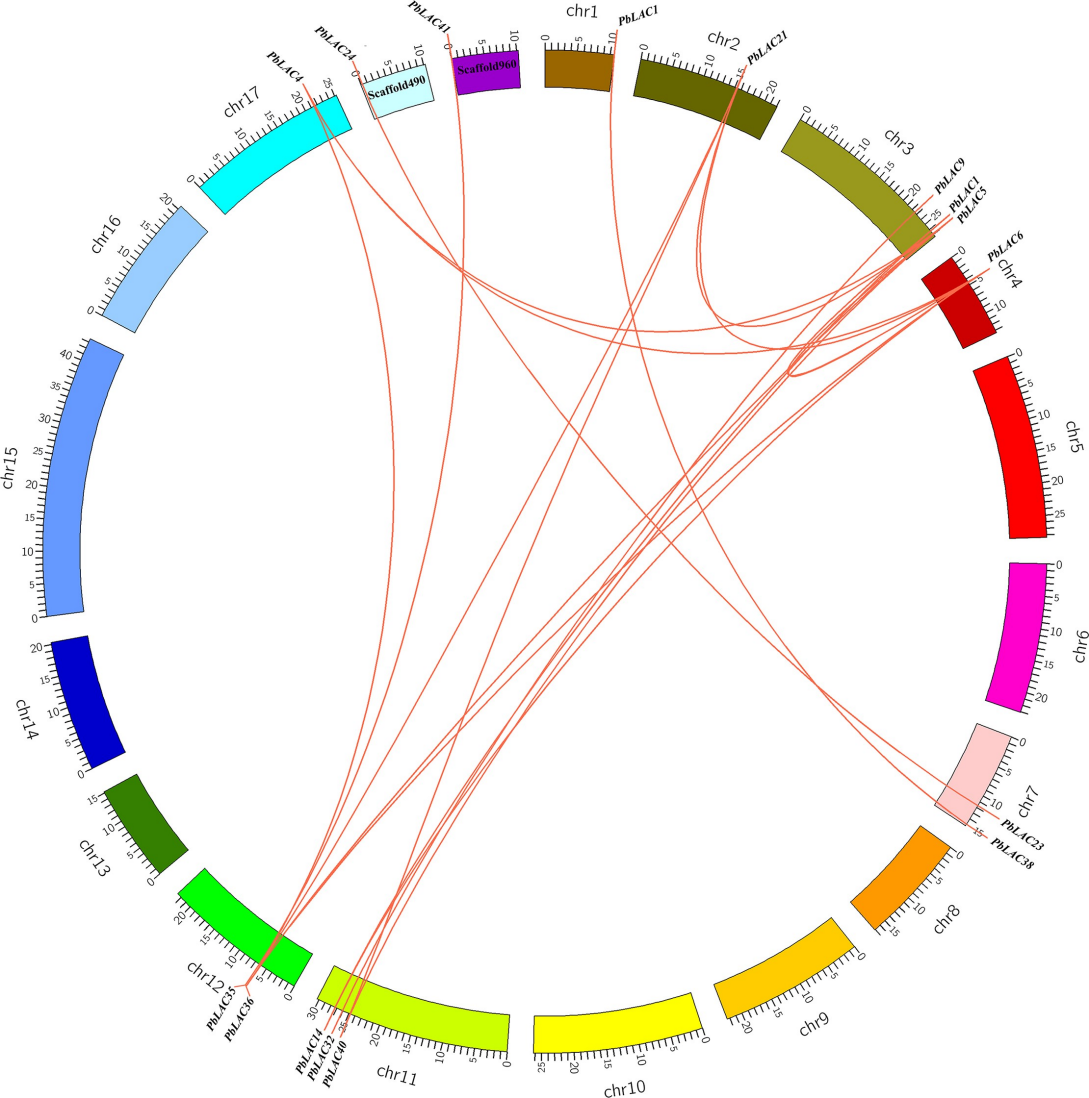
Segmental duplication gene pairs in the *PbL*AC family. The 17 chromosomes of pear are marked by different colours and labelled with their names (Chr1 to Chr17) on the outside. The duplicated gene pairs are connected by orange lines.

A total of 18 gene pairs of the *PbLAC* family participated in the gene duplication event (Fig 2, S3 Fig), and 16 gene pairs were found to exhibit segmental duplication events, which indicates that the expansion of the *PbLAC* family in the pear genome was mainly due to segmental duplication events.

The Ka (synonymous mutation) and Ks (non-synonymous mutation) values are usually used to represent the evolutionary pressure on a gene. In general, Ka/Ks>1 indicates positive selection, Ka/Ks = 1 indicates neutral selection, and Ka/Ks<1 indicates purification [30,39]. We analysed the Ka/Ks ratios of the 18 identified gene pairs (S6 Table) and found that the Ka/Ks ratios of all duplicated genes were less than 1, which indicated that these genes had undergone purification after duplication. In particular, the Ka/Ks ratios for *PbLAC4*/*PbLAC6*, *PbLAC4*/*PbLAC36*, *PbLAC1*/*PbLAC38* and *PbLAC6*/*PbLAC21*, which are pairs of duplicated genes, were less than 0.1, demonstrating strong purification. To evaluate the selection pressure in the duplicated region, we investigated the Ka/Ks ratio in a sliding window (S4 Fig), and the results showed that the Ka/Ks ratios of the three characteristic domains of laccase were mostly low, indicating that the conserved domain of PbLACs was strongly purified.

### Analysis of interspecies collinearity

To extensively understand the evolution and collinearity of the *LAC* family between different species, we also identified the 45, 43 and 54 members of the *LAC* family in the *P*. *persica*, *P*. *mume* and *F*. *vesca* genomes (S7 Table, S5 Fig). Pear, peach, mei and strawberry belong to the Rosaceae family and share a common ancient hexaploid ancestor with Arabidopsis [11,12]. Therefore, we analysed the collinearity between the laccase gene families of these five species.

**Fig 3:**
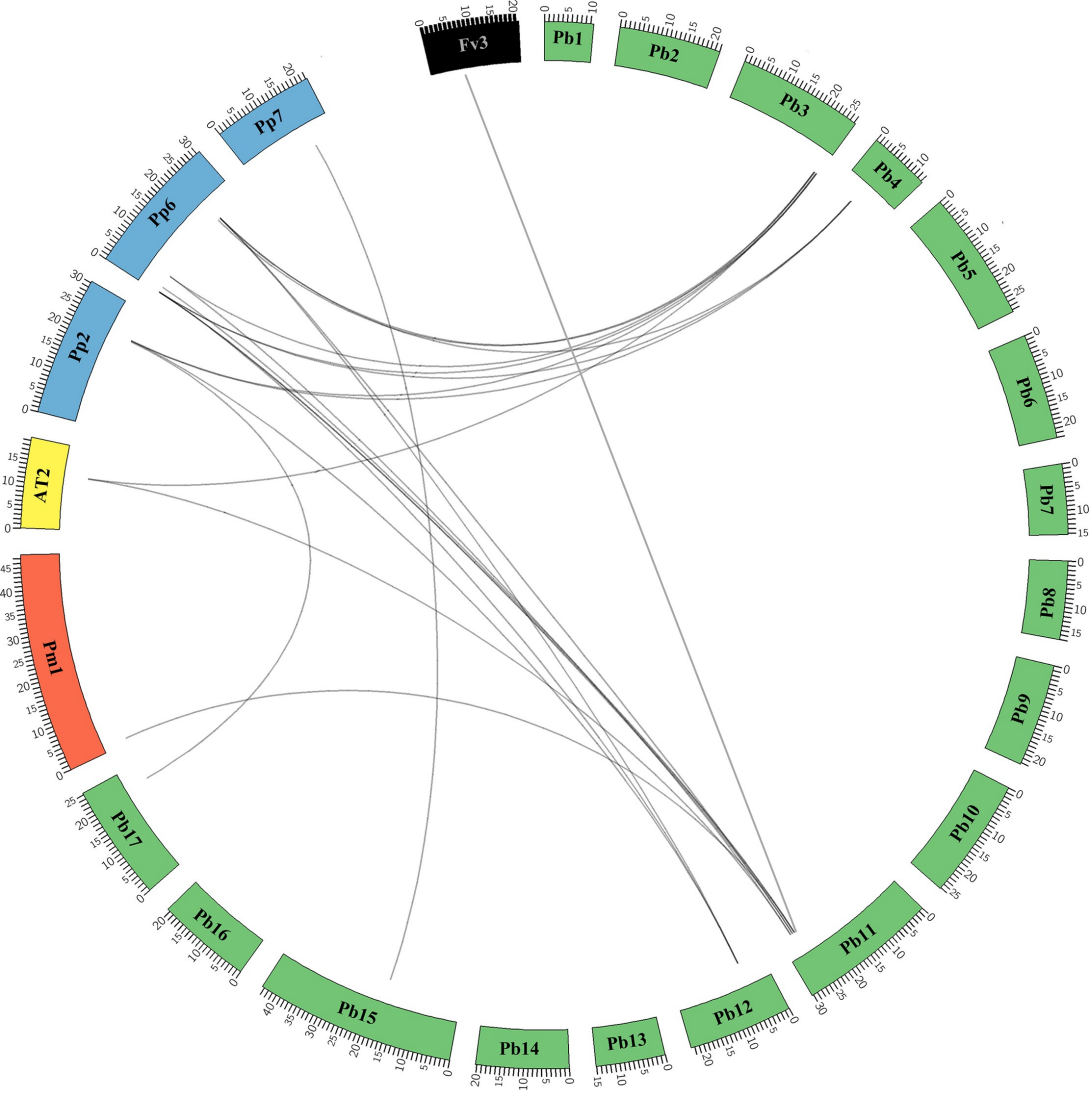
Collinearity analysis of *LAC* regions across *P*. *bretschneideri* (Pb), *P*. *mummer* (Pm), *P*. *persica* (Pp), *F*. *vesca* (Fv) and Arabidopsis (At). The chromosomes of each species are represented by different colours, and the chromosome numbers are indicated. The collinear gene pairs are connected by black lines.

For the *LAC* family, 25 collinear gene pairs were identified among pear, peach, mei and Arabidopsis (Fig 3, S8 Table), and these included two collinear gene pairs between pear and Arabidopsis, one pair between pear and mei, three pairs between pear and strawberry, and 19 pairs between pear and peach. Notably, *PbLAC36* (Pbr035962.1) forms collinear gene pairs with *PpLAC*, *PmLAC* and *FvLAC*, which indicated that these genes appeared before the divergence of the common ancestor of pear, mei, peach and strawberry. Interestingly, we found that many *PbLAC*s have collinear relationships with several *PpLAC*s at the same time. For example, ppa003646m, ppa022440m and ppa027203m have collinear relationships with *PbLAC5* (Pbr042315.1), and ppa003646m, ppa022440m and ppa027203m have collinear relationships with *PbLAC6* (Pbr012358.1). This finding suggested that they probably belong to paralogous gene pairs.

### Function prediction of *PbLAC* proteins in each phylogenetic group

**Fig 4:**
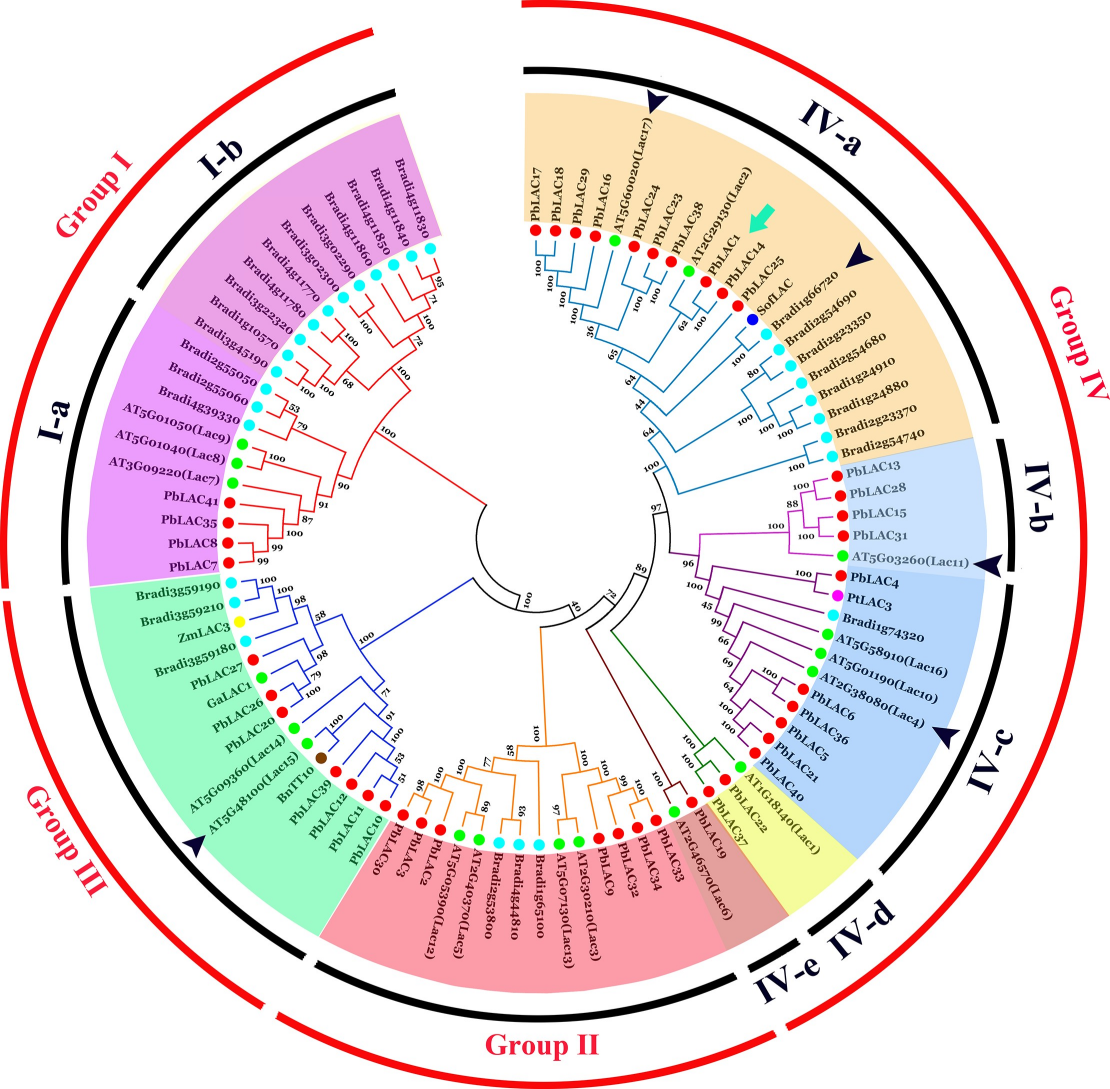
Phylogenetic tree of *LAC* protein sequences of various plants. Green arrows show PbLAC1 and PbLAC14. Black arrows show lignin-specific BdLAC and AtLACs (previously published). Red diamonds represent members of the *PbLAC* family.

We predicted the potential functions of *PbLAC* proteins through phylogenetic clustering (Fig 4). The 29 reported laccases of *B*. *distachyon* and the 17 reported laccases of Arabidopsis were used to construct an interspecies phylogenetic tree. Laccases that are associated with monolignols or flavonoid metabolites in five other species, including *B*. *napus* Transparent Testa10 (BnTT10), GaLAC1 of *G*. *arboretum*, SofLAC of *Saccharum* spp., PtLAC3 of *P*. *trichocarpa* and ZmLAC3 of *Zea mays* [17,40–42], were also used to construct the phylogenetic trees.

As clearly shown in Fig 4, the amino acid sequences of laccases in various species can be distributed into four phylogenetic groups (Group I-IV), and Group I can be further divided into two subgroups: Subgroup I-a and Subgroup I-b. Subgroup I-a consists of the LACs of *B*. *distachyon*, pear and Arabidopsis. Among these, AtLAC8 has been proven to affect the flowering time, and the functions of AtLAC9 and AtLAC7 are unknown [43]. All the LACs in Subgroup I-b belong to the *B*. *distachyon LAC* family, and none of these members have been shown to be involved in lignin synthesis [17]. In conclusion, none of the LACs clustered in Group I have been definitively associated with lignin metabolism, and similarly, no Group II members have yet been associated with lignin synthesis. Therefore, the *PbLAC*s in these two phylogenetic groups might not catalyse the polymerization of lignin monomers.

AtLAC15 and BnTT10 in Group III are mainly responsible for the synthesis of flavonoids [19,42], and PbLAC10, 11, 12, and 39 are clustered into one class and presumably have similar functions. In addition, three PbLACs (PbLAC20, 26 and 27) cluster with GaLAC1 [44], but their low identity and similarity to GaLAC1 (S9 Table) suggest that their functions might have changed.

Lignin-specific LACs are mostly clustered in Group IV, and according to the classification of AtLACs by Zhao et al. (2013) [22], this phylogenetic group can be further divided into five subgroups (Subgroup IV-a, Subgroup IV-b, Subgroup IV-c, Subgroup IV-d and Subgroup IV-e) (Fig 4). AtLAC17 and ten PbLACs (PbLAC1, 14, 16, 17, 18, 23, 24, 25, 29 and 38), AtLAC11 and four PbLACs (PbLAC13, 15, 28 and 31), and AtLAC4 and five PbLACs (PbLAC5, 6, 21, 36, 40) are clustered into Subgroup IV-a, Subgroup IV-b and Subgroup IV-c, respectively. More importantly, the sequence identity and similarity of 12 PbLACs (PbLAC1, 5, 6, 13, 15, 16, 21, 28, 29, 31, 36 and 40) and the AtLACs in their clusters (AtLAC4, AtLAC11, AtLAC17) are greater than 70% (S9 Table). These results indicate that these PbLACs might participate in lignin biosynthesis in pear.

Subgroup IV-d is composed of AtLAC1, PbLAC22 and PbLAC37, and Group IV-e is composed of AtLAC6 and PbLAC19. However, the functions of AtLAC1 and 6 are unclear [43], and the functions of these three PbLACs are therefore also unclear.

### Analysis of the spatiotemporal expression patterns of *PbLAC*s

**Fig 5:**
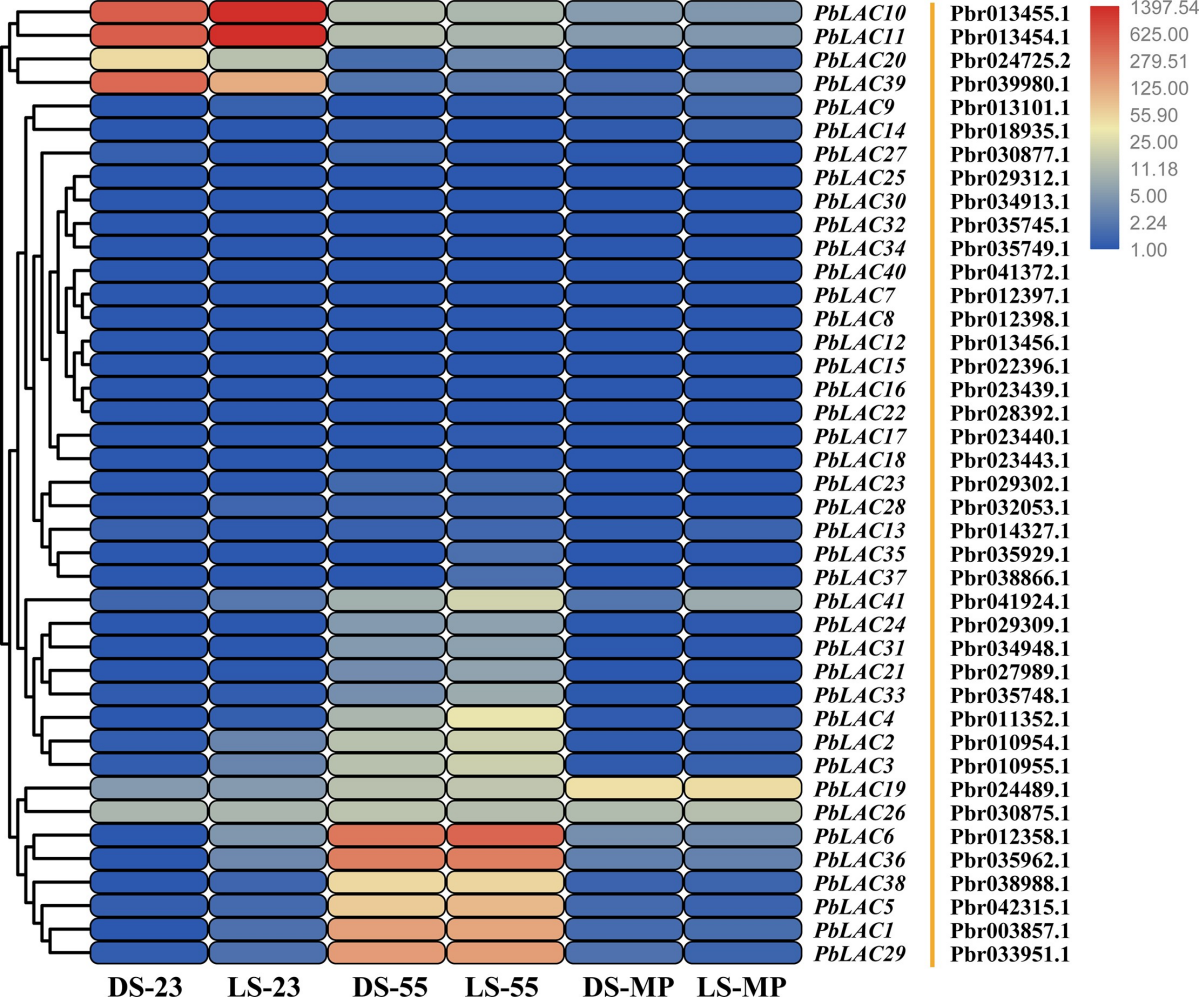
Expression profiles of *PbLAC*s in pear fruit at various developmental stages. The fragments per kilobase million (FPKM) values were obtained by RNA-sequencing analysis and are presented as a heat map. A colour scale is provided with the heat map to indicate the levels of differential expression. Yellow indicates a high level of expression, black signifies a medium level of expression, and purple denotes a low level of expression. DS: *Pyrus bretschneideri* cv. ‘Dangshan Su’. LS: *Pyrus bretschneideri* cv. ‘Lianglizaosu’.

To further screen for *PbLAC* members that play a major role in lignin synthesis in pear fruit, we analysed the expression patterns of the *PbLAC* family in pear fruit at three developmental stages (23 DAF, 55 DAF and at maturity) based on transcriptome sequencing data from *Pyrus bretschneideri* cv. ‘Dangshan Su’ (DS) and *Pyrus bretschneideri* cv. ‘Lianglizaosu’ (LS) (Fig 5). The reliability of the transcriptome sequencing data was verified by Zhang et al. (2017) by qRT-PCR [2]. The FPKM values for each *PbLAC* are listed in S10 Table.

Previous studies revealed that the stone cell and lignin contents showed a rise-fall tendency during the development of pear fruit and peaked at 55 DAF [2]. Notably, the expression levels of six *PbLAC*s (*PbLAC1*, *5*, *6*, *29*, *36* and *38*) showed a similar tendency to the stone cell and lignin contents in pear fruit, which suggesting that these genes are likely to be involved in lignin polymerization and stone cell formation in pear fruit. Among them, the transcript levels of *PbLAC1*, *PbLAC6*, *PbLAC29* and *PbLAC36* were significantly increased at 55 DAF, indicating the possibility that these four genes play a major role in stone cell development and lignin biosynthesis. To further clarify the temporal and spatial expression patterns of *PbLAC1*, *PbLAC6*, *PbLAC29* and *PbLAC36*, the expression levels of these four *PbLAC*s at eight developmental stages of fruit development and in different organs of the pear tree were studied. In addition, five *PbLAC*s (*PbLAC14*, *16*, *17*, *18* and *PbLAC25*) classified into the same group were selected for comparative purposes. The relevant parameters of each *PbLAC* qRT-PCR primer are listed in S11 Table.

**Fig 6:**
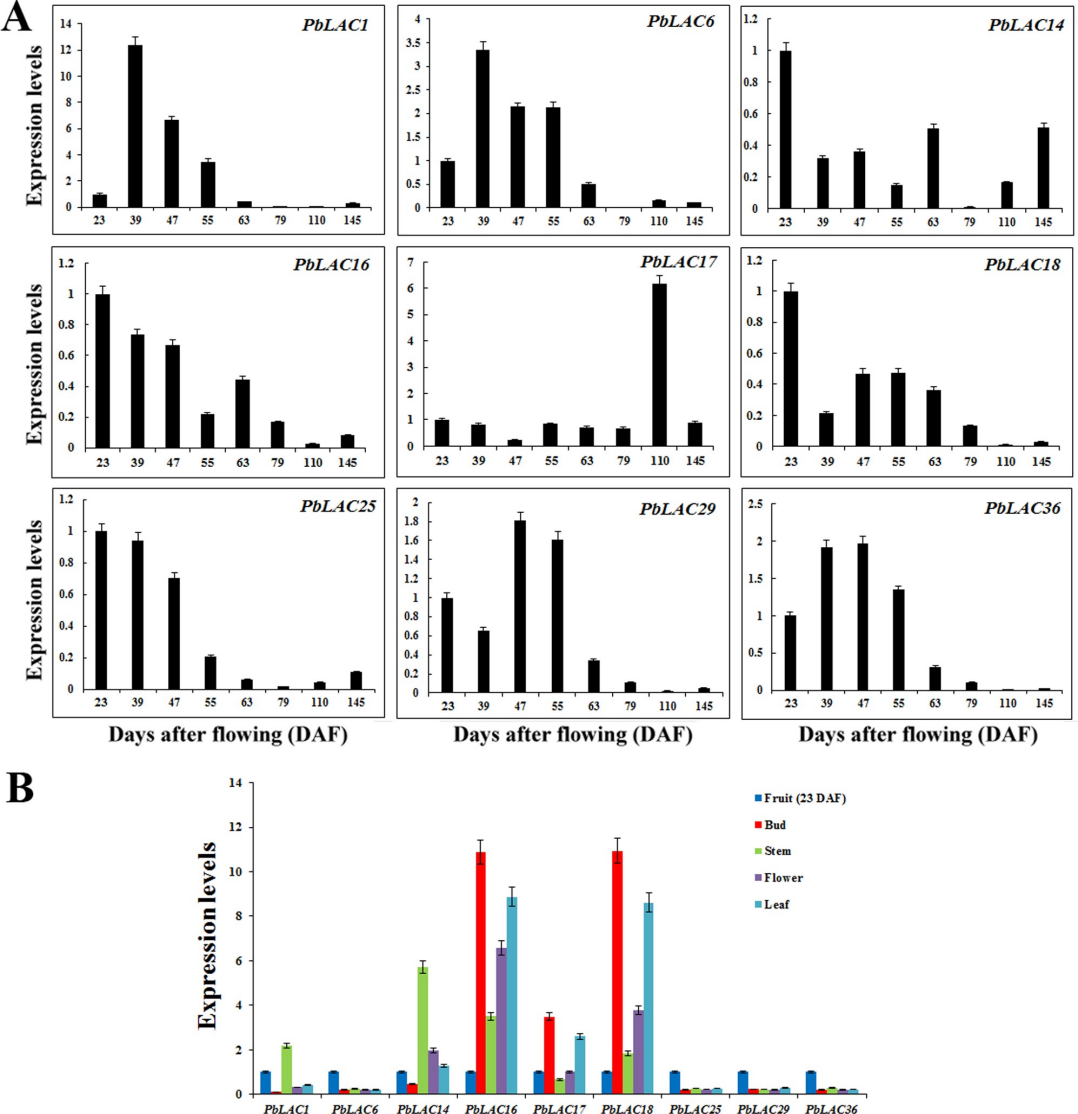
Spatiotemporal expression patterns of *PbLAC* genes. (A) Analysis of *PbLAC* expression patterns in pear fruit at various developmental stages. (B) Analysis of *PbLAC* expression patterns in various tissues of pear. The ordinate represents the relative expression levels of the genes, and the abscissa represents various members of the *PbLAC* family.

In agreement with the phylogenetic analysis, the expression patterns of *PbLAC1*, *PbLAC6*, *PbLAC29* and *PbLAC36* were consistent with the trends in the changes in the lignin and stone cell contents (Fig 6A). *PbLAC1* and *PbLAC14* are a pair of duplicated genes and are both target genes of *Pyr-miR1890* but exhibit different expression patterns in fruits. The expression of *PbLAC1* reached a peak at 39 DAF and decreased gradually after 63 DAF. However, the expression of *PbLAC14* at various developmental stages of fruit is irregular.

*PbLAC16*, *17* and *18* exhibit close relationships with *PbLAC29* and *AtLAC17*. As shown in Fig 6A, *PbLAC17* is present at a very low expression level in the early and middle stages (23–79 DAF), and its expression peaked at 110 DAF. The expression of *PbLAC16* and *18* decreased gradually starting from the early stage of fruit development. *PbLAC25* was highly expressed in fruit at 23–47 DAF, which in combination with its low expression level from 55 DAF to 145 DAF indicated that this laccase plays a major role in the early stage of fruit development. These four genes did not show the same trend as the lignin and stone cell contents in fruit during the corresponding period, which indicates that these genes might not participate in lignin synthesis and stone cell formation in fruit.

We also analysed the tissue-specific expression patterns of *PbLAC*s (Fig 6B). These *PbLAC*s were highly expressed in at least one of five organs (fruits, buds, stems, leaves and flowers). The expression levels of *PbLAC6*, *25*, *29* and *36* are higher in fruit than in other organs, which suggests that they might play important roles in pear fruit.

*PbLAC1* was found to be mainly expressed in stems and fruits. The expression of *PbLAC14* was higher in the stems, flowers and leaves than in fruits, which shows that *PbLAC1* and *14* also play important roles in organs other than fruits. *PbLAC16* and *18* showed their lowest expression in fruits and were highly expressed in four other organs. In particular, the expression levels of *PbLAC16*, *17* and *18* in leaves and buds were significantly higher than those in other organs, which suggests that these genes are associated with the growth and development of buds and leaves.

### Overexpression of *PbLAC1* in Arabidopsis increased the lignin content

Our previous studies revealed that *Pyr-miR1890* (homologous gene of *Ptr-miR397a*) can regulate the expression levels of *PbLAC1* and *PbLAC14* and might thus affect lignin metabolism and stone cell development [13]. This study also showed that *PbLAC1* has higher identity and similarity with AtLAC17, and the expression trend is consistent with the trends in lignin and stone cell content in fruits. However, a different expression trend was found for the expression of *PbLAC14*. Based on this analysis, the *PbLAC1* and *PbLAC14* genes were selected as candidates for further comparative investigation. We successfully cloned the *PbLAC1* and *14* CDS from pear complementary DNA (cDNA), and the encoded amino acid sequences are highly consistent with those of the lignin-specific LACs of other species, particularly three conserved laccase domains (S6 Fig).

**Fig 7:**
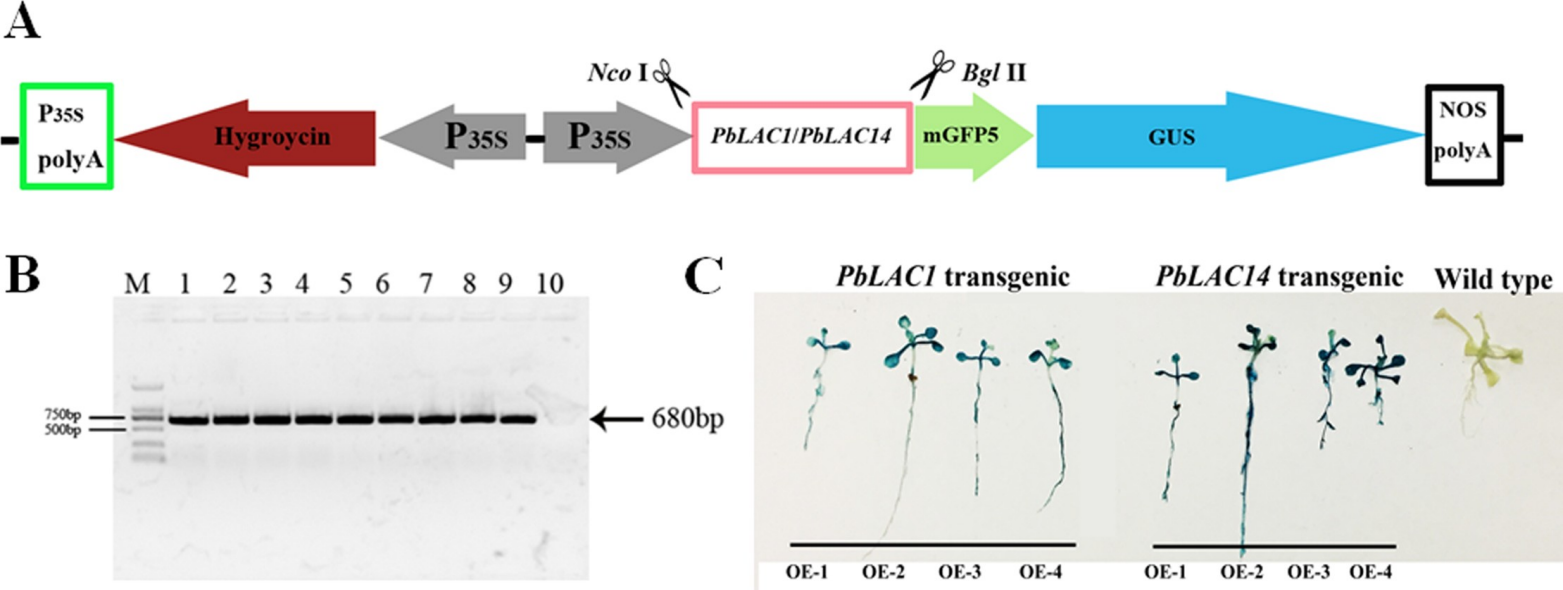
Overexpression of *PbLAC1* and *PbLAC14* in *Arabidopsis*. (A) pCAMBIA1304-*PbLAC1*/*PbLAC14*. (B) RT-PCR analysis for detecting green fluorescent protein (g*fp*) genes in transgenic *Arabidopsis*. M: DL2000 DNA Marker; 1: WT; 1–4: *PbLAC1* transgenic lines; 5–8: *PbLAC1* transgenic lines; 9: pCAMBIA1304; 10: water. (C) β-Glucuronidase (GUS) histochemical staining of transgenic Arabidopsis.

**Fig 8:**
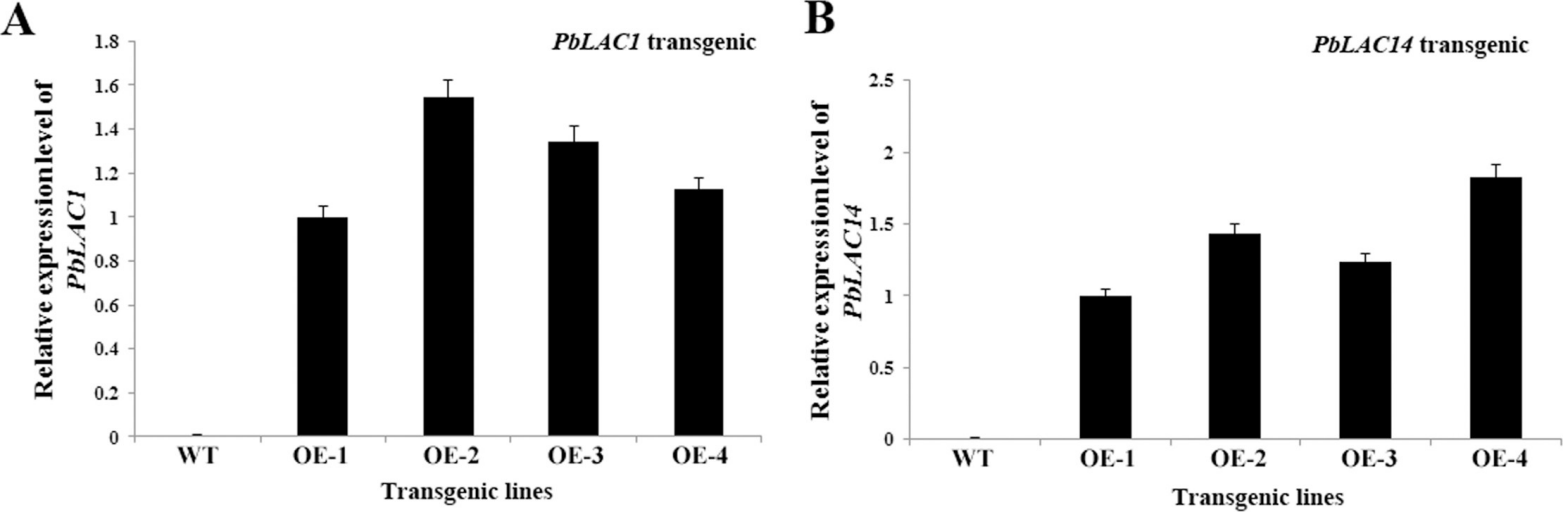
Expression analysis of transgenic *Arabidopsis thaliana* plants. (A) *PbLAC1* expressed in four transgenic lines and WT; (B) *PbLAC14* expressed in four transgenic lines and WT.

To further investigate the roles of *PbLAC1* and *PbLAC14* in lignin synthesis, we constructed eukaryotic expression vectors (Fig 7A). Specific primers designed with *gfp* on the pCAMBIA1304 vector were used to amplify the transgenic line DNA. The target fragments of approximately 700 bp were cloned successfully, which indicated that the exogenous genes were successfully integrated into the genome of Arabidopsis (Fig 7B). GUS staining showed that all lines showed obvious chromogenic reactions, indicating the successful transcription and translation of exogenous genes in Arabidopsis (Fig 7C). We subsequently successfully obtained four T_3_ generation transgenic lines that expressed *PbLAC1* and *PbLAC14*. As demonstrated by qRT-PCR analysis, the target gene (*PbLAC1*/*PbLAC14*) in the overexpression lines *PbLAC1*-OE2 and *PbLAC14*-OE4 was found to show the highest transcription level, followed by *PbLAC1*-OE3 and *PbLAC14*-OE2 (Fig 8). Therefore, *PbLAC1-*OE2, *PbLAC1*-OE3, *PbLAC14*-OE2 and *PbLAC14-*OE4 were selected for further study.

**Fig 9:**
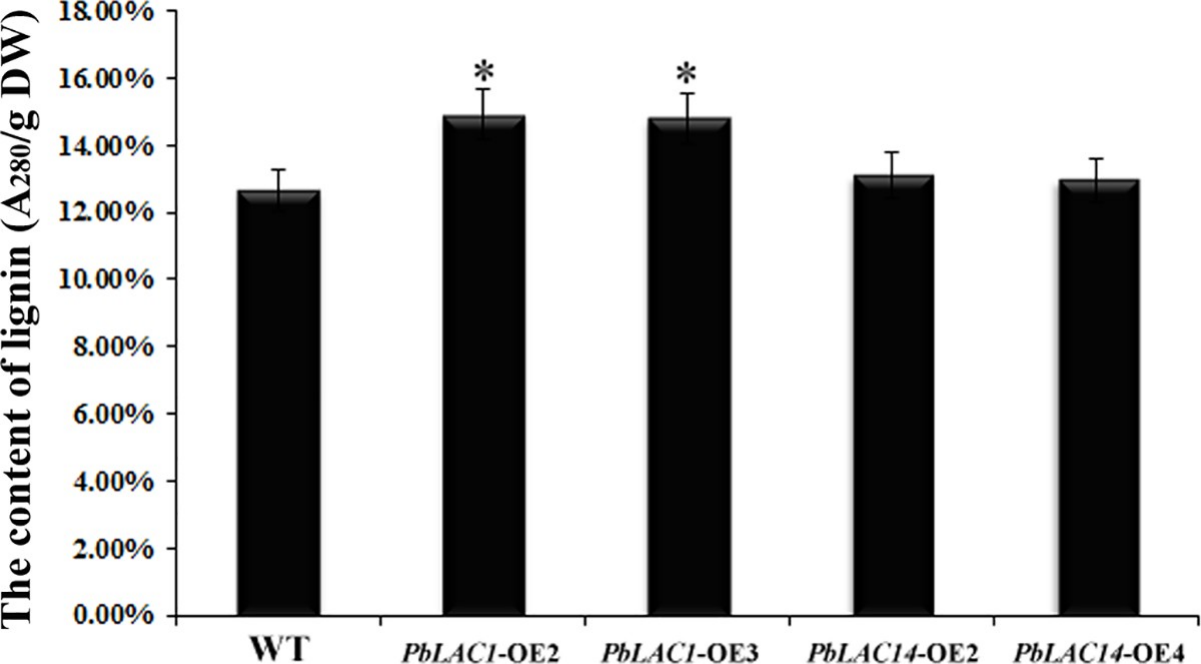
Determination of lignin content in inflorescence stem of Arabidopsis. *Significant difference in lignin content levels between WT and overexpression transgenic Arabidopsis (*P* < 0.05). The error bars indicate the standard errors from three biological replicates. DW: dry weight.

We used the acetyl bromide method to determine the lignin content in Arabidopsis inflorescence stems. The results showed that the lignin contents of *PbLAC1-*OE2 (14.90%) and *PbLAC1*-OE3 (14.80%) were higher than that of the WT (12.67%). The lignin contents of *PbLAC14-*OE2 and *PbLAC14-*OE4 were 13.11% and 12.98%, respectively, and these values showed no significant difference compared with that of the WT (Fig 9).

**Fig 10:**
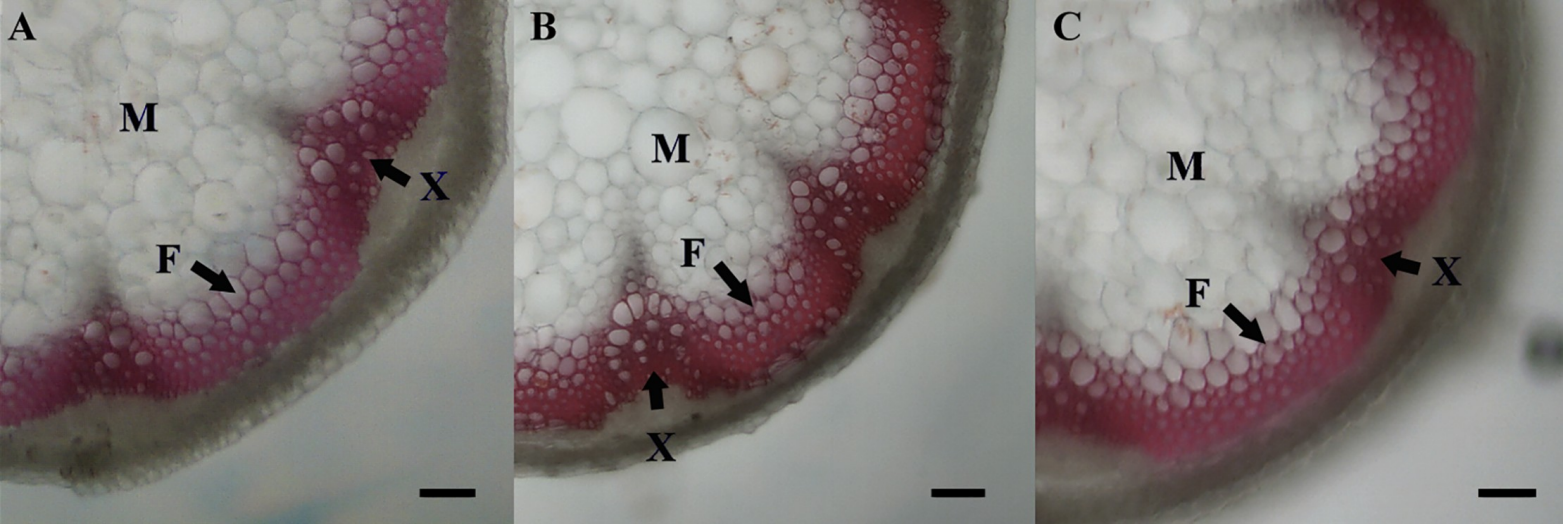
Wiesner staining of cross-sections of Arabidopsis inflorescence stem. All Arabidopsis plants were planted in the same environment, and the inflorescence stems were obtained from Arabidopsis plants grown for two months. (A) WT plants. (B) *PbLAC1*-overexpressing transgenic plants. (C) *PbLAC14*-overexpressing transgenic plants, bar = 25 μm. F: interfascicular fibre; X: xylem; M: medullary parenchyma.

**Fig 11:**
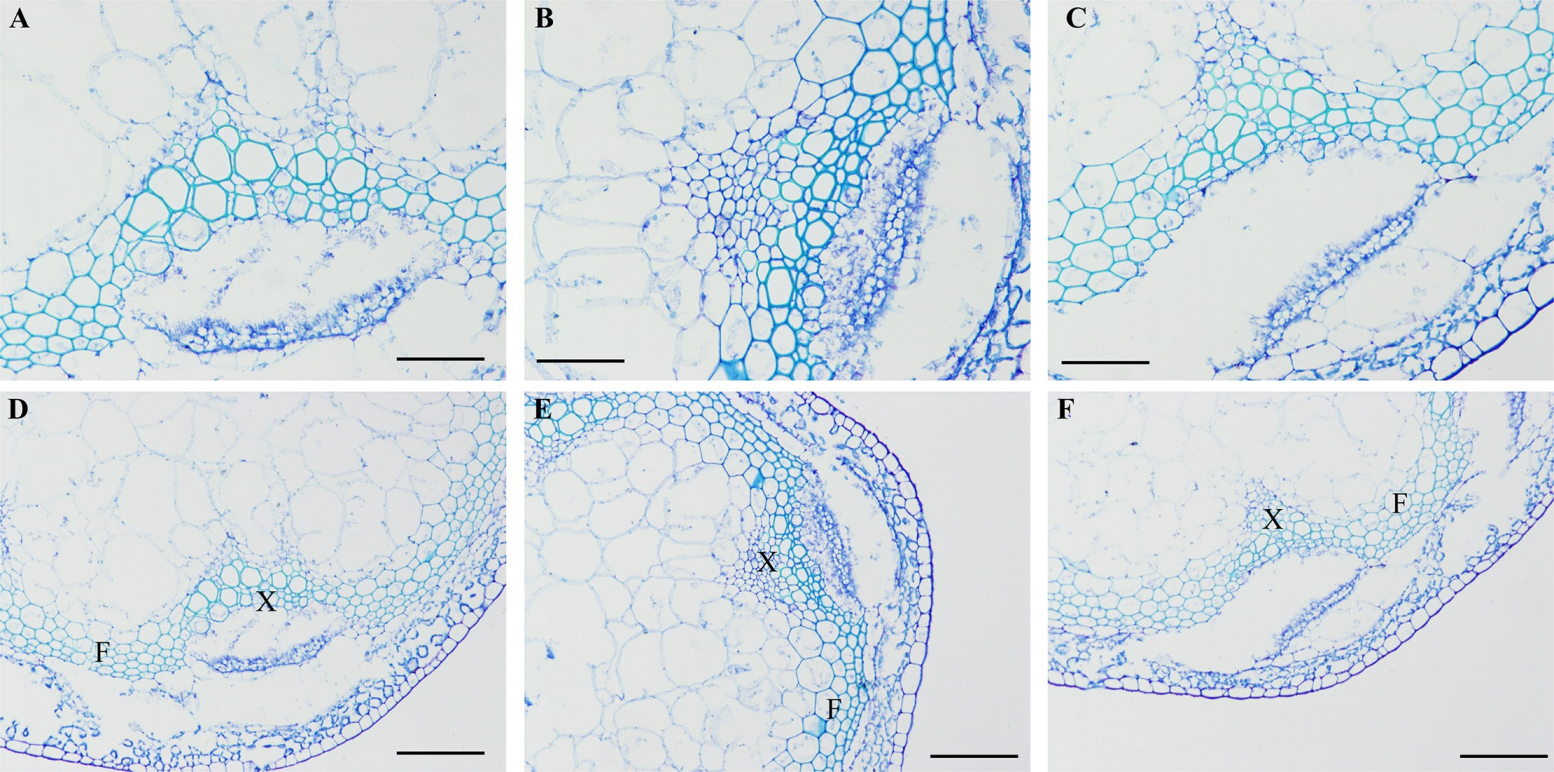
Toluidine blue staining of the inflorescence stems from WT and transgenic lines. (A) and (D) WT plants; (B) and (E) *PbLAC1*-overexpressing transgenic plants; (C) and (F) *PbLAC14*-overexpressing transgenic plants. (A)-(C): bar = 50 μm. (D)- (F): bar = 100 μm. F: interfascicular fibre; X: xylem.

Subsequently, we selected *PbLAC1-*OE2 and *PbLAC14-*OE2, which exhibited the highest lignin content, for histochemical staining to observe the phenotypic differences in the transverse section of the inflorescence stems. The Wiesner staining (phloroglucinol-HCl) results revealed that the xylem and interfascicular fibre of the *PbLAC1-*OE2 inflorescence stems showed the strongest staining. However, the staining of the xylem and interfascicular fibre region of the inflorescence stem of *PbLAC14* transgenic plants was similar to that of the WT plants (Fig 10). In addition, the toluidine blue straining showed the cell wall of the cross-sectional region of the inflorescence stem of Arabidopsis (Fig 11), and the cross-sections obtained from WT and *PbLAC14*-overexpressing transgenic plants showed no significant difference in the cell wall morphology of the xylem and the interfascicular fibre in the inflorescence stems. Notably, the comparisons of cross-sections of inflorescence stems from *PbLAC1*-overexpressing transgenic and WT plants revealed a significant increase in cell wall thickness in both the interfascicular fibre and xylem of the *PbLAC1* transgenic plants. These results indicated that the lignin accumulation and cell wall thickness of the interfascicular fibre and xylem cells in the *PbLAC1* transgenic plants were higher (by approximately 17% compared with the wild-type level) than those of the WT and *PbLAC14* transgenic plants.

**Fig 12:**
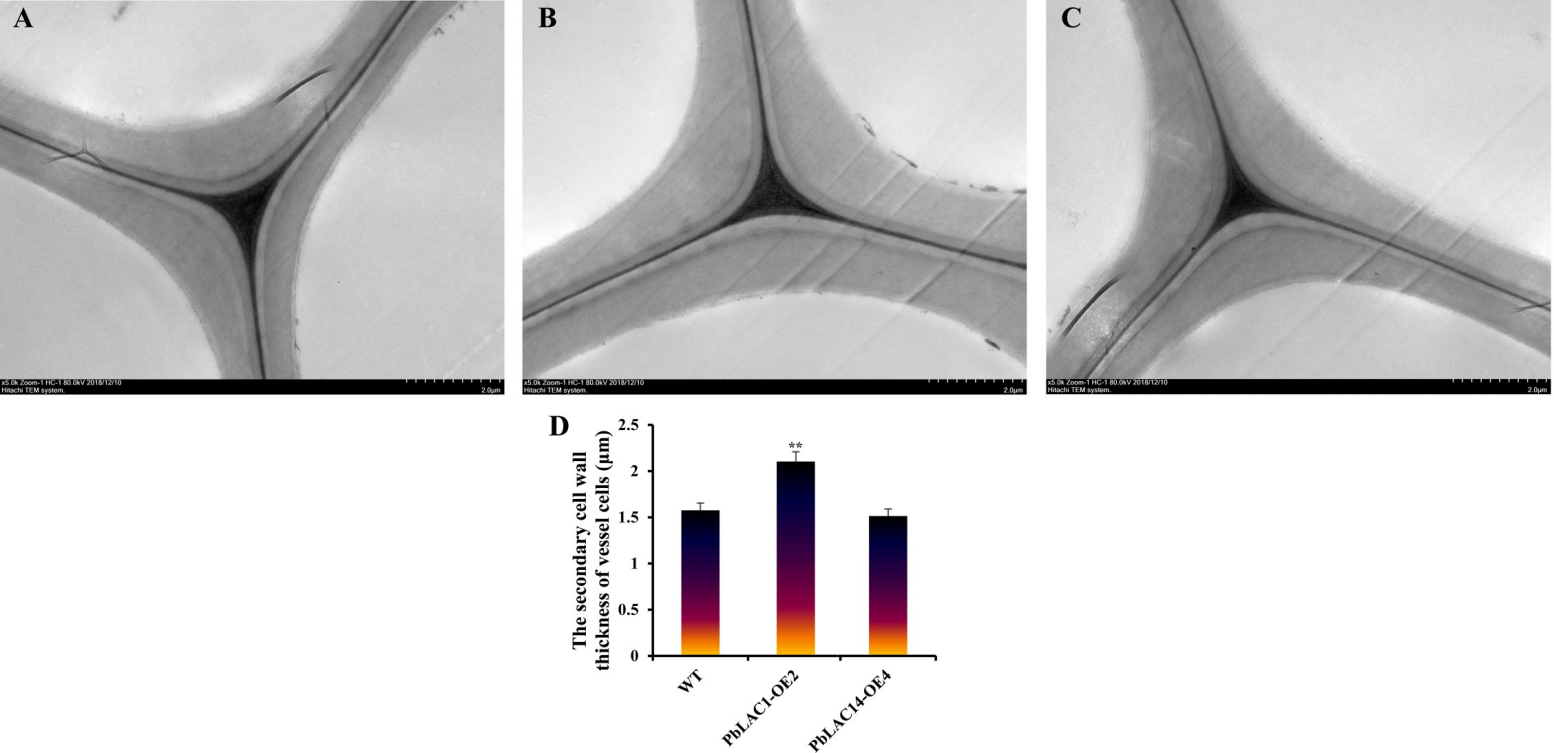
Ultramicroscopic observation of cell walls in the inflorescence stems of WT and transgenic lines. TEM images of the ultrastructure of the cell wall. (A) WT plants; (B) *PbLAC1*-overexpressing transgenic plants; (C) *PbLAC14*-overexpressing transgenic plants; (D) Statistical analysis of the secondary cell wall thickness of vessel cells in WT and transgenic plants. ** Significant difference between the secondary cell wall thickness of the WT and transgenic plants (*P* < 0.01).

To further clarify the role of *PbLAC1* and *PbLAC14* in cell wall development, the ultramicroscopic observation of the inflorescence stems of three genotypes of *Arabidopsis thaliana* (WT plants, *PbLAC1-*overexpressing transgenic plants and P*bLAC14-*overexpressing transgenic plants) were observed by TEM (Fig 12). The TEM observation and cell wall thickness measurements revealed that the cell wall thickness of the transgenic *PbLAC1* lines was significantly higher than those of the WT plants and transgenic *PbLAC14* lines (Fig 12). However, the difference in cell thickness between the *PbLAC14* transgenic lines and the WT plants was not significant. These results revealed that *PbLAC1* plays a key role in lignin synthesis and cell wall development, which is consistent with the bioinformatics results, whereas the overexpression of *PbLAC14* in Arabidopsis did not significantly increase the lignin content and cell wall thickness.

## Discussion

The content and size of stone cells are the most important factors affecting fruit quality [2,9,10]. Stone cells not only affect the texture and taste of the flesh but also are negatively correlated with the contents of various nutrients. The content of lignin in the mature stone cells of pear is 20–30% [8,45]. The differentiation of the parenchyma cells of the flesh into stone cells causes the secondary cell walls to thicken and induce the deposition of a large amount of lignin [6,7,16]. Therefore, the development of stone cells is closely related to the synthesis and deposition of lignin. Laccases are responsible for the polymerization of lignin monomers and play an important role in the formation of secondary cell walls [14,22]. Laccases exist in the form of gene families in plants, and their members are numerous and functional [17,46]. Therefore, the screening and identification of PbLACs associated with lignin synthesis are important for the regulation of lignin synthesis and stone cell development in pear.

We analysed the classification, conserved domains and phylogenetic relationships of PbLACs to better understand their role in lignin synthesis. In this study, we identified 41 members of the *PbLAC* family in pear, and this value is higher than the numbers of members in rice (30), *Arabidopsis* (17) and *B*. *distachyon* (29) but lower than the numbers of members in in *Populus* (49) [17,18,23,46]. Similar to those found in other plants, all PbLACs have three copper ion-binding sites [18], and PbLACs are mostly secretory proteins that are transported to the apoplast after synthesis and can catalyse the oxidation and polymerization of lignin monomers.

The 41 laccase members of pear can be divided into four phylogenetic groups, similarly to those of the *AtLAC* family [46]. An analysis of the phylogenetic tree of the *AtLAC* and *BdLAC* family members revealed that both AtLACs and BdLACs were present in each phylogenetic group, and the clustering results were consistent with those of previous studies [17], which indicated the reliability of the constructed phylogenetic tree. AtLAC17 in Subgroup IV-a, AtLAC11 in Subgroup IV-b and AtLAC4 in Subgroup IV-c have been shown to be related to lignin synthesis [22], which suggests that lignin-specific PbLACs are likely concentrated in these three subgroups. Therefore, the phylogenetic tree and sequence similarity analysis suggests that PbLAC1, 5, 6, 13, 15, 16, 21, 28, 29, 31, 36 and 40 are likely associated with lignin synthesis (Fig 4, S9 Table).

Previous studies have indicated that the stone cells of ‘Dangshan Su’ pear form between 23 and 67 DAF and that their content peaks at 55 DAF [2,5]. Our results revealed that the changes in the expression of *PbLAC1*, *PbLAC6*, *PbLAC29* and *PbLAC36* at different developmental stages of fruit were consistent with this dynamic trend (Fig 6A), which suggests that these four *PbLAC*s might play an important role in fruit lignin synthesis and stone cell development. Interestingly, we found that the expression levels of *PbLAC6*, *25*, *29*, and *36* in fruit are notably higher than those in other tissues, indicating the existence of tissue-specific promoters for these genes (Fig 6B).

Many studies have shown that miRNA can regulate *LAC* and thus affect lignin synthesis [23,24,47]. Our previous study revealed that *Pyr-miR1890* can regulate the expression of *PbLAC1* and *14*, which might in turn regulate pear fruit lignin metabolism and stone cell development [13]. Xue et al. (2018) also demonstrated that the overexpression of *PbrmiR397a* (also known as *Pyr-miR1890*) in tobacco significantly reduces the secondary cell wall thickness and lignin content of the plants [36]. Although *Pyr-miR1890* is currently known to regulate lignin biosynthesis by laccases, the specific biological functions of PbLAC in pears are unclear. To this end, this study focused on the role of the two target genes (*PbLAC1* and *14*) of *Pyr-miR1890* in lignin synthesis and cell wall development. A three-dimensional structural analysis showed that the three-dimensional structure of PbLAC1 is similar to those of BdLAC5 and SofLAC, but a higher similarity was found between PbLAC14 and AtLAC11 (S7 Fig). To verify their real functions, we analysed the roles of *PbLAC1* and *PbLAC14* in lignin synthesis through an overexpression analysis, and the results showed that the overexpression of *PbLAC1*, but not *PbLAC14*, in Arabidopsis could increase the lignin content and cell wall thickness of plants, which is consistent with the results predicted by us and Xue et al. (2018) [36]. A similar phenomenon has been observed in the laccase family of *B*. *distachyon* (*BdLAC* family). BdLAC5 and BdLAC6 show higher sequence identity and similarity with lignin-specific laccases and are both located in lignifying interfascicular fibres. However, the lignin content in the stem of the BdLAC6-deficient mutant was not significantly different from that of the WT, whereas the lignin content in the BdLAC5-deficient mutant was reduced by 10%.

In *Arabidopsis thaliana*, no significant lignin content changes were detected in the *Atlac11* single mutant, and the lignin content was slightly decreased in the double mutants *Atlac4 lac11* and *Atlac4 lac17*. However, the lignin content was significantly decreased in the *Atlac4 lac11 lac17* triple mutant, indicating functional redundancy among the genes [20,22,41]. PbLAC14 might show some similarities to AtLAC11. Specifically, PbLAC14 might exhibit low enzyme activity and therefore cannot cause a significant increase in lignin; alternatively, a compensatory effect might exist between *PbLAC1* and *PbLAC14* in pear fruit. It is also possible that *PbLAC14* is less closely related to lignin metabolism and has other biological functions. In future research, we will transform laccase gene mutants into *Arabidopsis* to further analyse their function in lignin synthesis.

## Conclusions

In conclusion, we screened and identified laccase family members in the pear genome, and the characteristics and evolution of the *PbLAC* family were systematically analysed. An expression pattern analysis revealed that *PbLAC1*, *PbLAC6*, *PbLAC29* and *PbLAC36* might be lignin-specific *PbLAC*s in pear fruit, and a heterologous expression analysis in Arabidopsis clearly showed that *PbLAC1* is involved in lignin metabolism and cell wall development. Thus, this study not only provides target genes for regulating the metabolism of pear lignin but also lays the foundation for clarifying the function of the *PbLAC* family.

## Supporting information

S1 Table Amino acid sequence information used for the construction of phylogenetic trees and sequence alignments. (XLS)

S2 Table Primers for qRT-PCR and gene cloning. (DOCX)

S3 Table Identification and characterization of the PbLACs in pear. (XLSX)

S4 Table Top 10 of 20 motifs and functional annotations. (DOCX)

S5 Table Numbers of *cis*-elements in the promoter regions of *PbLAC*s. (DOCX)

S6 Table Ka/Ks analysis of *PbLAC* duplicated genes from pear. (DOCX)

S7 Table List of LAC protein sequences of three Rosaceae species. (XLSX)

S8 Table Sequence identity and similarity among pear and *LAC* protein sequences of various plants. (DOCX)

S9 Table Sequence identity and similarity among pear and *LAC* protein sequences of various plants. (DOCX)

S10 Table FPKM values of *PbLAC*s in pear fruit at various developmental stages. (DOCX)

S11 Table The relevant parameters of each PbLAC qRT-PCR primer. (DOCX)

S1 Fig Gene structures and conserved motifs of PbLACs based on evolutionary relationships. (A) Phylogenetic relationships of PbLACs. (B) Distribution of 20 putative conserved motifs in PbLAC proteins. (C) Exon-intron organization of *PbLAC*s.
(TIF)

S2 Fig Chromosomal locations and gene duplications of *PbLAC*s on 13 chromosomes. (TIF)

S3 Fig Microsynteny regions of the *LAC* family in *Pyrus bretschneideri*. The green bars represent chromosomes, and he chromosome types and regions are shown on the right. The numbers on both sides of the chromosome are the suffixes of the Genome ID of each gene. Homologous gene pairs are connected by straight lines, and the *LAC* lines are red. The blue lines indicate other anchor genes in the region, and non-homologous genes are shown in white.
(TIF)

S4 Fig Sliding window plots of duplicated *PbLAC*s. Grey, blue and purple blocks indicate the positions of Cu-oxidase_3 domain, Cu-oxidase domain and Cu-oxidase_2 domain, respectively. The window size was 150 bp, and the step size was 9 bp. The x-axis represents the nucleotide position of the gene pair, and the y-axis represents the Ka/Ks ratio.
(TIF)

S5 Fig Phylogenetic analysis of LACs from pear, peach, mei, strawberry and Arabidopsis. An N-J tree with 41 pear, 54 strawberry, 43 mei, 45 peach and 17 Arabidopsis LAC proteins was created using MEGA5.0.
(TIF)

S6 Fig Multiple sequence alignment of LAC proteins of various plants. The three consecutive grey-shaded areas are the three conserved domains, namely, CuRO_1_LCC_Plant, CuRO_2_LCC_Plant and CuRO_3_LCC_Plant.
(TIF)

S7 Fig Predicted three-dimensional structures of LACs from pear and other species. AtLAC4, AtLAC11, AtLAC 17, BdLAC5 and SofLAC have been proven to be responsible for lignin biosynthesis.
(TIF)

## References

[1] Cheng X, Li M, Li D, Zhang J, Jin Q, Sheng L, et al Characterization and analysis of *CCR* and *CAD* gene families at the whole-genome level for lignin synthesis of stone cells in pear (*Pyrus bretschneideri*) fruit. Biol Open. 2017;6: 1602–1613. 10.1242/bio.026997 29141952 PMC5703608

[2] Zhang J, Cheng X, Jin Q, Su X, Li M, Yan C, et al Comparison of the transcriptomic analysis between two Chinese white pear (*Pyrus bretschneideri* Rehd.) genotypes of different stone cells contents. PLoS One. 2017;12: 1–22. 10.1371/journal.pone.0187114 29088238 PMC5663431

[3] Choi J, Choi J, Hong K, Kim W. Cultivar differences of stone cells in pear flesh and their effects on fruit quality. Hortic Environ Biotechnol. 2007;48: 17–31.

[4] Choi J, Lee S. Distribution of stone cell in Asian, Chinese, and European pear fruit and its morphological changes. J Appl Bot Food Qual. 2013;189: 185–189. 10.5073/JABFQ.2013.086.025

[5] Cai Y, Li G, Nie J, Lin Y, Nie F, Zhang J, et al Study of the structure and biosynthetic pathway of lignin in stone cells of pear. Sci Hortic (Amsterdam). Elsevier B.V.; 2010;125: 374–379. 10.1016/j.scienta.2010.04.029

[6] Jin Q, Yan C, Qiu J, Zhang N, Lin Y, Cai Y. Structural characterization and deposition of stone cell lignin in Dangshan Su pear. Sci Hortic (Amsterdam). Elsevier B.V.; 2013;155: 123–130. 10.1016/j.scienta.2013.03.020

[7] Zhao SG, Zhang JG, Zhao YP, Zhang YX. New discoveries of stone cell differentiation in fruitlets of “Yali” pears (*Pyrus bretschneideri* Rehd.). J Food, Agric Environ. 2013;11: 937–942.

[8] Brahem M, Renard CMGC, Gouble B, Bureau S, Le Bourvellec C. Characterization of tissue specific differences in cell wall polysaccharides of ripe and overripe pear fruit. Carbohydr Polym. Elsevier Ltd.; 2017;156: 152–164. 10.1016/j.carbpol.2016.09.019 27842809

[9] Yan C, Yin M, Zhang N, Jin Q, Fang Z, Lin Y, et al Stone cell distribution and lignin structure in various pear varieties. Sci Hortic (Amsterdam). Elsevier B.V.; 2014;174: 142–150. 10.1016/j.scienta.2014.05.018

[10] Li N, Ma Y, Song Y, Tian C, Zhang L, Li L. Anatomical studies of stone cells in fruits of four different pear cultivars. Int J Agric Biol. 2017; 610–614. 10.17957/IJAB/15.0304

[11] Cheng X, Su X, Muhammad A, Li M, Zhang J, Sun Y, et al Molecular characterization, evolution, and expression profiling of the *Dirigent* (*DIR*) family genes in Chinese white pear (*Pyrus bretschneideri*). Front Genet. 2018;9: 1–15. 10.3389/fgene.2018.00001 29713336 PMC5911567

[12] Wu J, Wang Z, Shi Z, Zhang S, Ming R, Zhu S, et al The genome of the pear (*Pyrus bretschneideri* Rehd.). Genome Res. 2013;23: 396–408. 10.1101/gr.144311.112 23149293 PMC3561880

[13] Cheng X, Yan C, Zhang J, Ma C, Li S, Jin Q, et al The effect of different pollination on the expression of Dangshan Su pear microRNA. Biomed Res Int. 2017; 2017: 2794040 10.1155/2017/2794040 28497043 PMC5402243

[14] Schuetz M, Benske A, Smith RA, Watanabe Y, Tobimatsu Y, Ralph J, et al Laccases direct lignification in the discrete secondary cell wall domains of protoxylem. Plant Physiol. 2014;166: 798–807. 10.1104/pp.114.245597 25157028 PMC4213109

[15] Yi Chou E, Schuetz M, Hoffmann N, Watanabe Y, Sibout R, Samuels AL. Distribution, mobility, and anchoring of lignin-related oxidative enzymes in Arabidopsis secondary cell walls. J Exp Bot. 2018;69 10.1093/jxb/ery067 29481639 PMC6018803

[16] Barros J, Serk H, Granlund I, Pesquet E. The cell biology of lignification in higher plants. Ann Bot. 2015;115: 1053–1074. 10.1093/aob/mcv046 25878140 PMC4648457

[17] Wang Y, Bouchabke-Coussa O, Lebris P, Antelme S, Soulhat C, Gineau E, et al LACCASE5 is required for lignification of the *Brachypodium distachyon* culm. Plant Physiol. 2015;168: 192–204. 10.1104/pp.114.255489 25755252 PMC4424014

[18] Liu Q, Luo L, Wang X, Shen Z, Zheng L. Comprehensive analysis of rice laccase gene (*OsLAC*) family and ectopic expression of *OsLAC10* enhances tolerance to copper stress in Arabidopsis. Int J Mol Sci. 2017;18 10.3390/ijms18020209 28146098 PMC5343771

[19] Pourcel L, Routaboul JM, Kerhoas L, Caboche M, Lepiniec L, Debeaujon I. *Transparent Testa10* encodes a laccase-like enzyme involved in oxidative polymerization of flavonoids in Arabidopsis seed coat. Plant Cell. 2005;17: 2966–2980. 10.1105/tpc.105.035154 16243908 PMC1276023

[20] Berthet S, Demont-Caulet N, Pollet B, Bidzinski P, Cezard L, Le Bris P, et al Disruption of LACCASE4 and 17 results in tissue-specific alterations to lignification of *Arabidopsis thaliana* stems. Plant Cell. 2011;23: 1124–1137. 10.1105/tpc.110.082792 21447792 PMC3082258

[21] Balasubramanian VK, Rai KM, Thu SW, Hii MM, Mendu V. Genome-wide identification of multifunctional laccase gene family in cotton (*Gossypium* spp.); Expression and biochemical analysis during fiber development. Sci Rep. 2016;6: 1–16. 10.1038/s41598-016-0001-8 27679939 PMC5041144

[22] Zhao Q, Nakashima J, Chen F, Yin Y, Fu C, Yun J, et al LACCASE is necessary and nonredundant with PEROXIDASE for lignin polymerization during vascular development in Arabidopsis. Plant Cell. 2013;25: 3976–3987. 10.1105/tpc.113.117770 24143805 PMC3877815

[23] Lu S, Li Q, Wei H, Chang MJ, Tunlaya-Anukit S, Kim H, et al *Ptr-miR397a* is a negative regulator of laccase genes affecting lignin content in *Populus trichocarpa*. Proc Natl Acad Sci. 2013;110: 10848–10853. 10.1073/pnas.1308936110 23754401 PMC3696765

[24] Wang CY, Zhang S, Yu Y, Luo YC, Liu Q, Ju C, et al *MiR397b* regulates both lignin content and seed number in Arabidopsis via modulating a laccase involved in lignin biosynthesis. Plant Biotechnol J. 2014;12: 1132–1142. 10.1111/pbi.12222 24975689

[25] Tamura K, Peterson D, Peterson N, Stecher G, Nei M, Kumar S. MEGA5: Molecular evolutionary genetics analysis using maximum likelihood, evolutionary distance, and maximum parsimony methods. Mol Biol Evol. 2011;28: 2731–2739. 10.1093/molbev/msr121 21546353 PMC3203626

[26] Liu F, Xu Y, Jiang H, Jiang C, Du Y, Gong C, et al Systematic identification, evolution and expression analysis of the Zea mays *PHT1* gene family reveals several new members involved in root colonization by arbuscular mycorrhizal fungi. Int J Mol Sci. 2016;17: 1–18. 10.3390/ijms17060930 27304955 PMC4926463

[27] Wang Q, Liu J, Wang Y, Zhao Y, Jiang H, Cheng B. Systematic analysis of the maize PHD-finger gene family reveals a subfamily involved in abiotic stress response. Int J Mol Sci. 2015;16: 23517–23544. 10.3390/ijms161023517 26437398 PMC4632711

[28] Lin YX, Jiang HY, Chu ZX, Tang XL, Zhu SW, Cheng BJ. Genome-wide identification, classification and analysis of heat shock transcription factor family in maize. BMC Genomics. 2011;12: 76 10.1186/1471-2164-12-76 21272351 PMC3039612

[29] Lee TH, Tang H, Wang X, Paterson AH. PGDD: A database of gene and genome duplication in plants. Nucleic Acids Res. 2013;41: 1152–1158. 10.1093/nar/gks1104 23180799 PMC3531184

[30] Lin Y, Cheng Y, Jin J, Jin X, Jiang H, Yan H, et al Genome duplication and gene loss affect the evolution of heat shock transcription factor genes in legumes. PLoS One. 2014;9 10.1371/journal.pone.0102825 25047803 PMC4105503

[31] Livak KJ, Schmittgen TD. Analysis of relative gene expression data using real-time quantitative PCR and the 2^-ΔΔCT^ method. Methods. 2001;25: 402–408. 10.1006/meth.2001.1262 11846609

[32] Clough SJ, Bent AF. Floral dip: A simplified method for Agrobacterium-mediated transformation of *Arabidopsis thaliana*. Plant J. 1998;16: 735–743. 10.1046/j.1365-313X.1998.00343.x 10069079

[33] Pradhan Mitra P, Loqué D. Histochemical staining of *Arabidopsis thaliana* secondary cell wall elements. J Vis Exp. 2014; 1–11. 10.3791/51381 24894795 PMC4186213

[34] Anderson NA, Tobimatsu Y, Ciesielski PN, Ximenes E, Ralph J, Donohoe BS, et al manipulation of guaiacyl and syringyl monomer biosynthesis in an Arabidopsis cinnamyl alcohol dehydrogenase mutant results in atypical lignin biosynthesis and modified cell wall structure. Plant Cell. 2015;27: 2195–2209. 10.1105/tpc.15.00373 26265762 PMC4568507

[35] De Meester B, de Vries L, Özparpucu M, Gierlinger N, Corneillie S, Pallidis A, et al Vessel-specific reintroduction of CINNAMOYL COA REDUCTASE 1 (CCR1) in dwarfed *ccr1* mutants restores vessel and xylary fiber integrity and increases biomass. Plant Physiol. 2017;176: pp.01462.2017. 10.1104/pp.17.01462 29158331 PMC5761799

[36] Xue C, Yao JL, Qin MF, Zhang MY, Allan AC, Wang DF, et al *PbrmiR397a* regulates lignification during stone cell development in pear fruit. Plant Biotechnol J. 2018; 1–15. 10.1111/pbi.12950 29754465 PMC6330545

[37] Donà M, Macovei A, Faè M, Carbonera D, Balestrazzi A. Plant hormone signaling and modulation of DNA repair under stressful conditions. Plant Cell Rep. 2013;32: 1043–1052. 10.1007/s00299-013-1410-9 23508254

[38] Derksen H, Rampitsch C, Daayf F. Signaling cross-talk in plant disease resistance. Plant Sci. Elsevier Ireland Ltd; 2013;207: 79–87. 10.1016/j.plantsci.2013.03.004 23602102

[39] Zhang W, Yan H, Chen W, Liu J, Jiang C, Jiang H, et al Genome-wide identification and characterization of maize expansin genes expressed in endosperm. Mol Genet Genomics. 2014;289: 1061–1074. 10.1007/s00438-014-0867-8 25213600

[40] Wang J, Wang C, Zhu M, Yu Y, Zhang Y, Wei Z. Generation and characterization of transgenic poplar plants overexpressing a cotton laccase gene. Plant Cell Tissue Organ Cult. 2008;93: 303–310. 10.1007/s11240-008-9377-x

[41] Cesarino I, Araújo P, Sampaio Mayer JL, Vicentini R, Berthet S, Demedts B, et al Expression of *SofLAC*, a new laccase in sugarcane, restores lignin content but not S:G ratio of Arabidopsis *lac17* mutant. J Exp Bot. 2013;64: 1769–1781. 10.1093/jxb/ert045 23418623

[42] Zhang K, Lu K, Qu C, Liang Y, Wang R, Chai Y, et al Gene silencing of *BnTT10* family genes causes retarded pigmentation and lignin reduction in the seed coat of *Brassica napus*. PLoS One. 2013;8: 1–10. 10.1371/journal.pone.0061247 23613820 PMC3632561

[43] Cai X, Davis EJ, Ballif J, Liang M, Bushman E, Haroldsen V, et al Mutant identification and characterization of the laccase gene family in Arabidopsis. J Exp Bot. 2006;57: 2563–2569. 10.1093/jxb/erl022 16804053

[44] Wang GD, Li QJ, Luo B, Chen XY. *Ex planta* phytoremediation of trichlorophenol and phenolic allelochemicals via an engineered secretory laccase. Nat Biotechnol. 2004;22: 893–897. 10.1038/nbt982 15195102

[45] Lu G, Li Z, Zhang X, Wang R, Yang S. Expression analysis of lignin-associated genes in hard end pear (*Pyrus pyrifolia* Whangkeumbae) and its response to calcium chloride treatment conditions. J Plant Growth Regul. 2015;34: 251–262. 10.1007/s00344-014-9461-x

[46] McCaig BC, Meagher RB, Dean JFD. Gene structure and molecular analysis of the laccase-like multicopper oxidase (LMCO) gene family in *Arabidopsis thaliana*. Planta. 2005;221: 619–636. 10.1007/s00425-004-1472-6 15940465

[47] Huang JH, Qi YP, Wen SX, Guo P, Chen XM, Chen LS. Illumina microRNA profiles reveal the involvement of *miR397a* in *Citrus adaptation* to long-term boron toxicity via modulating secondary cell-wall biosynthesis. Sci Rep. Nature Publishing Group; 2016;6: 1–14. 10.1038/s41598-016-0001-8 26962011 PMC4790630

