# Correction: Comprehensive genome-wide analysis of the pear (*Pyrus bretschneideri*) laccase gene (*PbLAC*) family and functional identification of *PbLAC1* involved in lignin biosynthesis

**DOI:** 10.1371/journal.pone.0228183

**Published:** 2020-01-16

**Authors:** Xi Cheng, Guohui Li, Chenhui Ma, Muhammad Abdullah, Jinyun Zhang, Hai Zhao, Qing Jin, Yongping Cai, Yi Lin

In Fig 12, incorrect images are shown for panels A and B. The authors have provided a corrected version here.

**Fig 12:**
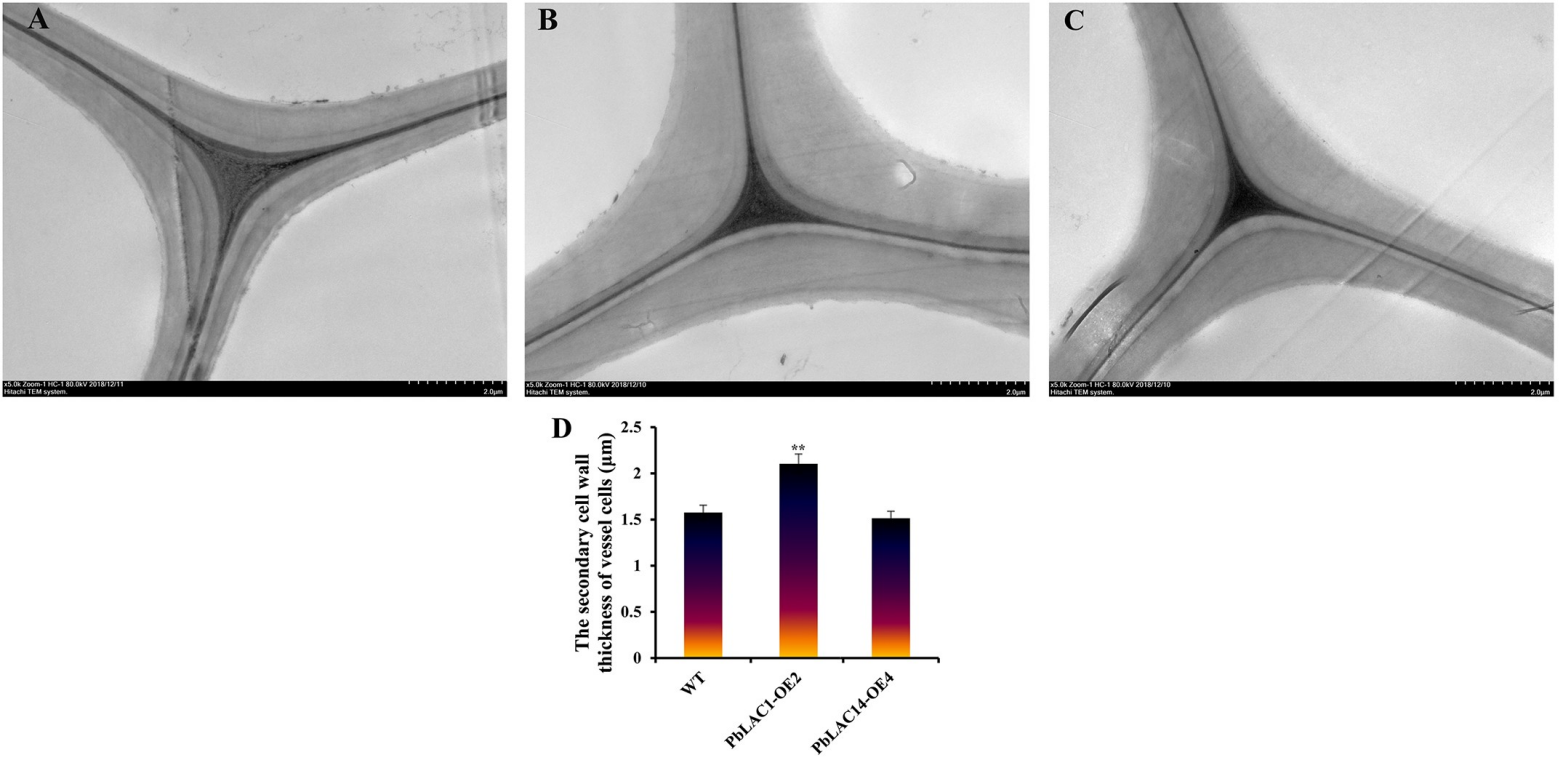
Ultramicroscopic observation of cell walls in the inflorescence stems of WT and transgenic lines. TEM images of the ultrastructure of the cell wall. (A) WT plants; (B) *PbLAC1*-overexpressing transgenic plants; (C) *PbLAC14*-overexpressing transgenic plants; (D) Statistical analysis of the secondary cell wall thickness of vessel cells in WT and transgenic plants. ** Significant difference between the secondary cell wall thickness of the WT and transgenic plants (*P* < 0.01).
