# Supplementary material for: Comprehensive genome-wide analysis of the pear (Pyrus bretschneideri) laccase gene (PbLAC) family and functional identification of PbLAC1 involved in lignin biosynthesis
Source: PLoS One. 2019 Feb 12;14(2):e0210892. doi: 10.1371/journal.pone.0210892 (PMC6372139; doi:10.1371/journal.pone.0210892)
Supplement: S2 Table — (DOCX) [file pone.0210892.s002.docx]

**Table S2 Primers for qRT-PCR and gene cloning.**

| **Name** | **Sense 5’-3’** | **Antisense 5’-3’** | **Amplicon size** | **Function** |
| --- | --- | --- | --- | --- |
| *Tubulin-q* | AGAACAAGAACTCGTCCTAC, | GAACTGCTCGCTCACTCTCC | 155 bp | qRT-PCR |
| *PbLAC1-q* | TGGCTTTTCTTCTTGCTCTTATCTC | CATTGCTGCAGCTGGCAGT | 68 bp |  |
| *PbLAC6-q* | CATTGCCATTTGGAAGTG | GATTGATCGAGGCTCCTT | 144 bp |  |
| *PbLAC14-q* | AGCCAATGTGCCCAAAACTG | GGCATGGGTTGGTTCCAA | 68 bp |  |
| *PbLAC16-q* | CTTGAAGATGGGCTGGGTAG | GTAATGGATTGAAAAGATCATCAGC | 105 bp |  |
| *PbLAC17-q* | ATACGATTCCTAGCAGATAACC | AGCCAAGCCATCTTCAAG | 89 bp |  |
| *PbLAC18-q* | TTCTTGCCGATAATCCAGGTGTGTG | GGGGGAGGGAGCAGTTTTTGATTGG | 127 bp |  |
| *PbLAC25-q* | GAAAGAGACGCAACCAAA | CACAAGAAATCTATGAGCAAAG | 80 bp |  |
| *PbLAC29-q* | GCTTCCCAACCAAAAGTT | TGTAGCTCAGATCGAGTCA | 75 bp |  |
| *PbLAC36-q* | TCTTGGTGGACAATGGTA | GAACAGAGGCTCCTTTTG | 95 bp |  |
| *PbLAC1-v* | CATGCCATGGCCATGGCGTCTTCTATTCCTTTTTCAC | GAAGATCTACACTTTGGGAGATCGGACGG |  | [Vector construction](http://www.baidu.com/link?url=6EgnmQyeVon-R0_YQrVAID65NX4dx6chUDrwya2hXsL9qVGFcQhHIq0DffrGz3ImLBME9bYpcmbx0E-sz8_DsTC64ueElP4tIvz4zRaC-Ay_0DlZqG6elo1RqBrMMWx7) |
| *PbLAC14-v* | CATGCCATGGCCATGGGGTCTGCGATTCCTTTG | GAAGATCTACACTTGGGAAGATCGGACGGT |  |  |
| *GFP* | GGAGAAGAACTTTTCACTGG | GTAATCCCAGCAGCTGTTAC |  | Molecular testing |
| *PbLAC1-T* | ACTCAGTGCCCTATTCAAACAAACCAGA | CGTTAATCGTATAGGCATTGGAGACATT |  |  |
| *PbLAC14-T* | ATACTAGTGTGGAATTGGTGATGCA | CATGAGCCAAACGCCTGGGTTGTCTGCA |  |  |
