# Supplementary material for: Comprehensive genome-wide analysis of the pear (Pyrus bretschneideri) laccase gene (PbLAC) family and functional identification of PbLAC1 involved in lignin biosynthesis
Source: PLoS One. 2019 Feb 12;14(2):e0210892. doi: 10.1371/journal.pone.0210892 (PMC6372139; doi:10.1371/journal.pone.0210892)
Supplement: S4 Table — (DOCX) [file pone.0210892.s004.docx]

**Table S4 Top 10 motifs of 20 and functional annotations.**

| **Name** | **Consensus Sequence** | **Functional annotations in NCBI** | **Functional annotations in Pfam** |
| --- | --- | --- | --- |
| **Motif 1** | 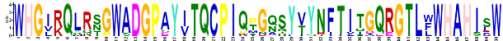 | cd13849,CuRO_1_LCC | PF07732,Multicopper oxidase |
| **Motif 2** | 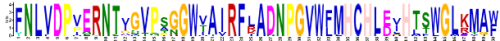 | cd13897,CuRO_3_LCC | PF07731,Multicopper oxidase |
| **Motif 3** | 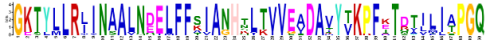 | cd13875,CuRO_2_LCC | PF00394,Multicopper oxidase |
| **Motif 4** | 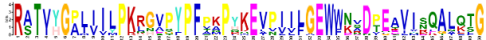 | TIGR03389, laccase_11, laccase  plant | PF07731,Multicopper oxidase |
| **Motif 5** | 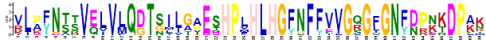 | TIGR03389, laccase_11, laccase  plant | PF07731,Multicopper oxidase |
| **Motif 6** | 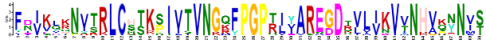 | TIGR03389, laccase_11, laccase  plant | PF07732,Multicopper oxidase |
| **Motif 7** | 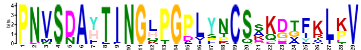 | TIGR03389, laccase_11, laccase plant. | PF00394,Multicopper oxidase |
| **Motif 8** | 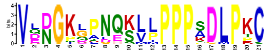 | TIGR03389, laccase_11, laccase plant. | /  / |
| **Motif 9** | 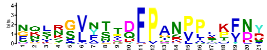 | TIGR03389, laccase_11, laccase laccase,plant. | /  / |
| **Motif 10** | 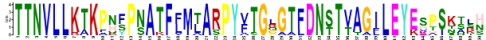 | TIGR03389, laccase_11, laccase | /  / |
