# Supplementary material for: Comprehensive genome-wide analysis of the pear (Pyrus bretschneideri) laccase gene (PbLAC) family and functional identification of PbLAC1 involved in lignin biosynthesis
Source: PLoS One. 2019 Feb 12;14(2):e0210892. doi: 10.1371/journal.pone.0210892 (PMC6372139; doi:10.1371/journal.pone.0210892)
Supplement: S5 Table — (DOCX) [file pone.0210892.s005.docx]

**Table S5 Numbers of *cis*-elements in promoter region of *PbLAC*s.**

| Element | MBS | MRE | AC | LTR | HSE | TC-rich repeat | ABRE | ERE | CGTCA motif | TCA-element | Total |
| --- | --- | --- | --- | --- | --- | --- | --- | --- | --- | --- | --- |
| Function | Drought stress | Light response |  | Low-temperature stress | Heat stress | Defense and stress | ABA response | Ethylene response | MeJA response | SA response |  |
| *PbLAC1* | 2 | 1 | 2 | 1 | 1 | 3 | 1 | 1 |  | 1 | 13 |
| *PbLAC2* | 1 |  |  | 1 |  |  | 6 |  | 3 | 1 | 12 |
| *PbLAC3* | 1 | 2 |  | 2 | 1 | 2 |  |  | 5 | 4 | 17 |
| *PbLAC4* | 3 |  |  | 2 |  | 1 |  |  | 3 | 1 | 10 |
| *PbLAC5* | 2 |  |  |  | 3 | 1 |  | 1 |  | 1 | 8 |
| *PbLAC6* | 2 |  | 2 |  |  | 2 |  |  | 2 | 2 | 10 |
| *PbLAC7* | 3 | 1 |  |  |  | 2 | 2 | 1 | 2 | 1 | 12 |
| *PbLAC8* | 3 | 1 |  |  |  | 2 | 2 | 1 | 2 | 1 | 12 |
| *PbLAC9* | 5 | 1 |  | 2 | 1 |  | 1 | 1 | 1 | 1 | 13 |
| *PbLAC10* |  |  |  | 2 | 1 | 1 | 2 |  | 5 | 4 | 15 |
| *PbLAC11* | 1 |  | 1 |  | 1 | 1 | 2 | 1 | 1 | 1 | 9 |
| *PbLAC12* |  |  |  |  | 1 | 1 | 1 | 1 | 3 | 1 | 8 |
| *PbLAC13* | 2 |  |  |  | 1 | 1 | 3 |  | 3 |  | 10 |
| *PbLAC14* | 1 | 1 |  |  | 2 |  | 1 |  | 1 | 2 | 8 |
| *PbLAC15* | 1 |  |  | 1 | 1 | 1 |  |  | 3 | 1 | 8 |
| *PbLAC16* | 3 |  |  |  | 1 | 1 | 1 |  |  | 3 | 9 |
| *PbLAC17* | 2 | 2 |  |  | 3 | 4 |  | 1 | 2 | 2 | 16 |
| *PbLAC18* | 2 | 2 |  |  | 3 | 3 |  | 1 | 2 | 2 | 15 |
| *PbLAC19* | 1 | 2 |  |  | 2 | 2 |  |  | 2 | 2 | 11 |
| *PbLAC20* | 3 | 1 |  |  | 1 | 1 | 3 |  |  |  | 9 |
| *PbLAC21* | 4 |  |  |  | 3 | 1 | 1 |  | 1 |  | 10 |
| *PbLAC22* | 1 |  | 3 |  | 1 | 2 | 1 | 5 | 1 | 1 | 15 |
| *PbLAC23* | 2 |  |  |  | 2 | 3 | 1 |  | 3 | 4 | 15 |
| *PbLAC24* | 2 |  |  |  | 2 | 2 | 1 |  | 2 | 4 | 13 |
| *PbLAC25* | 2 | 2 | 1 | 1 | 2 | 2 | 3 |  | 2 | 1 | 16 |
| *PbLAC26* | 2 | 2 |  | 1 | 1 | 3 | 2 |  | 2 | 1 | 14 |
| *PbLAC27* | 1 |  |  | 3 | 3 | 1 | 2 | 7 | 1 | 1 | 19 |
| *PbLAC28* | 3 | 1 |  | 1 | 1 |  | 4 |  | 4 | 1 | 15 |
| *PbLAC29* | 1 |  |  |  | 2 | 3 | 3 |  |  | 1 | 10 |
| *PbLAC30* | 1 | 2 |  | 2 | 1 | 2 |  |  | 5 | 4 | 17 |
| *PbLAC31* | 2 | 1 | 2 |  | 3 | 2 |  |  | 2 |  | 12 |
| *PbLAC32* | 2 | 1 |  | 1 | 2 |  | 2 |  | 2 |  | 10 |
| *PbLAC33* | 2 | 1 |  | 1 | 2 |  | 2 |  | 2 |  | 10 |
| *PbLAC34* | 2 | 1 |  | 1 | 2 |  | 2 |  | 2 |  | 10 |
| *PbLAC35* | 2 |  |  | 1 | 1 | 1 | 3 |  | 2 |  | 10 |
| *PbLAC36* |  | 1 |  | 1 | 1 | 1 |  |  |  | 1 | 5 |
| *PbLAC37* | 5 | 1 |  | 1 | 1 | 1 | 1 |  | 2 | 1 | 13 |
| *PbLAC38* | 2 |  |  |  | 2 | 3 | 1 |  | 3 | 4 | 15 |
| *PbLAC39* | 1 | 1 |  |  | 9 | 1 | 2 |  | 1 | 2 | 17 |
| *PbLAC40* | 1 |  |  |  | 1 | 1 | 2 |  | 1 |  | 6 |
| *PbLAC41* | 2 |  |  |  | 1 | 1 | 1 | 1 | 1 | 3 | 10 |
| Total | 78 | 28 | 11 | 25 | 66 | 59 | 59 | 22 | 79 | 60 | 487 |
