# Supplementary material for: Comprehensive genome-wide analysis of the pear (Pyrus bretschneideri) laccase gene (PbLAC) family and functional identification of PbLAC1 involved in lignin biosynthesis
Source: PLoS One. 2019 Feb 12;14(2):e0210892. doi: 10.1371/journal.pone.0210892 (PMC6372139; doi:10.1371/journal.pone.0210892)
Supplement: S6 Table — (DOCX) [file pone.0210892.s006.docx]

**Table S6** **Ka/Ks analysis for *PbLAC* duplicated genes from pear.**

| **Duplicated genes** | | **Ka** | **Ks** | **Ka/Ks** | **Purifying**  **selection** | **Duplicate**  **type** |
| --- | --- | --- | --- | --- | --- | --- |
| *PbLAC1* | *PbLAC14* | 0.0346 | 0.1651 | 0.2095 | yes | Segmental |
| *PbLAC6* | *PbLAC36* | 0.0328 | 0.1873 | 0.1751 | yes | Segmental |
| *PbLAC9* | *PbLAC32* | 0.0799 | 0.1875 | 0.4261 | yes | Segmental |
| *PbLAC21* | *PbLAC40* | 0.0079 | 0.0243 | 0.3251 | yes | Segmental |
| *PbLAC21* | *PbLAC5* | 0.0429 | 0.2040 | 0.2102 | yes | Segmental |
| *PbLAC23* | *PbLAC24* | 0.0432 | 0.0450 | 0.9600 | yes | / |
| *PbLAC35* | *PbLAC41* | 0.0739 | 0.4180 | 0.1767 | yes | / |
| *PbLAC40* | *PbLAC5* | 0.0337 | 0.1904 | 0.1769 | yes | Segmental |
| *PbLAC1* | *PbLAC38* | 0.2089 | 2.7849 | 0.0750 | yes | Segmental |
| *PbLAC4* | *PbLAC6* | 0.2575 | 2.7923 | 0.0922 | yes | Segmental |
| *PbLAC4* | *PbLAC36* | 0.2540 | 2.8031 | 0.0906 | yes | Segmental |
| *PbLAC4* | *PbLAC5* | 0.2628 | 1.9526 | 0.1345 | yes | Segmental |
| *PbLAC6* | *PbLAC21* | 0.1627 | 1.6339 | 0.0995 | yes | Segmental |
| *PbLAC11* | *PbLAC39* | 0.0777 | 0.1986 | 0.3912 | yes | Segmental |
| *PbLAC14* | *PbLAC38* | 0.5179 | 4.2635 | 0.1214 | yes | Segmental |
| *PbLAC21* | *PbLAC36* | 0.1689 | 1.5926 | 0.1060 | yes | Segmental |
| *PbLAC6* | *PbLAC5* | 0.1642 | 1.3580 | 0.1209 | yes | Segmental |
| *PbLAC36* | *PbLAC5* | 0.1682 | 1.3397 | 0.1255 | yes | Segmental |

Note: PbLAC24 and PbLAC41 are not localized to specific chromosomes, so it is impossible to determine the duplicate type.
