# Supplementary material for: Comprehensive genome-wide analysis of the pear (Pyrus bretschneideri) laccase gene (PbLAC) family and functional identification of PbLAC1 involved in lignin biosynthesis
Source: PLoS One. 2019 Feb 12;14(2):e0210892. doi: 10.1371/journal.pone.0210892 (PMC6372139; doi:10.1371/journal.pone.0210892)
Supplement: S8 Table — (DOCX) [file pone.0210892.s008.docx]

**Table S8 Orthologous gene pairs were identified in five species.**

| ***PbLIM* family** | **Orthologous gene pairs** | | | |  | |
| --- | --- | --- | --- | --- | --- | --- |
| **Pear** | **Peach** | **Strawberry** | **Mei** | **Arabidopsis** | |  |
| Pbr003857.1 | ppa003319m/ppa003296m |  |  | AT2G29130 | |  |
| Pbr042315.1 | [ppa003646m](http://chibba.agtec.uga.edu/duplication/index/details?lc=ppa003646m)/ppa022440m/ppa027203m |  |  |  | |  |
| Pbr012358.1 | ppa003646m/ppa022440m/ppa027203m |  |  |  | |  |
| [Pbr014327.1](http://chibba.agtec.uga.edu/duplication/index/details?lc=Pbr014327.1) | ppa003577m |  |  |  | |  |
| Pbr018935.1 | ppa003296m |  |  | AT2G29130 | |  |
| Pbr027989.1 | ppa022440m/ppa003646m |  |  |  | |  |
| Pbr035748.1 |  | mrna28184 |  |  | |  |
| Pbr035749.1 | ppa000767m | mrna28183 | Pm000175 |  | |  |
| Pbr035962.1 | ppa022440m/ppa003646m/ppa027203m |  |  |  | |  |
| Pbr038988.1 | ppa003319m |  |  |  | |  |
| Pbr041372.1 | ppa022440m |  |  |  | |  |
| Pbr041924.1 | ppa003587m | mrna18757 |  |  | |  |
