# Supplementary material for: Comprehensive genome-wide analysis of the pear (Pyrus bretschneideri) laccase gene (PbLAC) family and functional identification of PbLAC1 involved in lignin biosynthesis
Source: PLoS One. 2019 Feb 12;14(2):e0210892. doi: 10.1371/journal.pone.0210892 (PMC6372139; doi:10.1371/journal.pone.0210892)
Supplement: S9 Table — (DOCX) [file pone.0210892.s009.docx]

**Table S9 Sequence identity and similarity among pear and *LAC* protein sequences of various plants.**

| **Iden/Sim (%/%)** | **AtLAC4** | **AtLAC11** | **AtLAC17** | **AtLAC2** | **GaLAC1** |
| --- | --- | --- | --- | --- | --- |
| **PbLAC1** | 52.47/66.10 | 53.06/65.82 | 70.68/79.49 | 71.50/82.08 | - |
| **PbLAC5** | 74.20/82.21 | 59.68/71.76 | 53.86/66.21 | 53.38/67.59 | - |
| **PbLAC6** | 74.87/84.85 | 58.05/72.92 | 53.42/66.27 | 52.25/67.47 | - |
| **PbLAC13** | 57.85/70.90 | 76.28/84.78 | 53.49/65.42 | 52.07/66.72 | - |
| **PbLAC14** | 51.45/66.78 | 51.70/64.80 | 52.00/71.53 | 70.99/82.08 | - |
| **PbLAC15** | 59.93/73.23 | 75.44/85.59 | 52.23/66.27 | 50.87/66.09 | - |
| **PbLAC16** | 54.17/66.15 | 55.38/66.67 | 78.51/85.44 | 68.69/78.55 | - |
| **PbLAC17** | 45.75/56.29 | 45.59/56.27 | 66.27/73.59 | 57.14/65.31 | - |
| **PbLAC18** | 27.78/34.36 | 27.50/34.15 | 40.39/44.69 | 34.91/39.91 | - |
| **PbLAC20** | - | - | - | - | 54.72/69.23 |
| **PbLAC21** | 71.96/79.82 | 57.55/69.45 | 52.59/65.17 | 52.60/65.63 | - |
| **PbLAC23** | 47.77/61.62 | 48.01/59.97 | 63.39/71.47 | 60.93/72.09 | - |
| **PbLAC24** | 51.26/65.77 | 50.68/64.36 | 68.46/77.01 | 65.48/77.83 | - |
| **PbLAC25** | 56.12/68.37 | 54.08/67.52 | 66.89/77.87 | 63.88/75.47 | - |
| **PbLAC26** | - | - | - | - | 55.42/68.18 |
| **PbLAC27** | - | - | - | - | 37.74/52.83 |
| **PbLAC28** | 55.33/67.33 | 72.58/80.94 | 50.00/61.61 | 49.27/63.13 | - |
| **PbLAC29** | 51.87/63.41 | 51.79/63.47 | 74.88/83.20 | 64.66/74.27 | - |
| **PbLAC31** | 58.69/71.45 | 75.09/83.99 | 51.54/65.41 | 50.17/65.57 | - |
| **PbLAC36** | 74.69/83.78 | 59.47/73.10 | 53.60/66.10 | 52.77/67.82 | - |
| **PbLAC38** | 49.57/63.08 | 50.17/61.30 | 64.80/72.96 | 62.67/73.29 | - |
| **PbLAC40** | 74.29/82.50 | 58.61/71.58 | 53.44/65.98 | 53.21/66.90 | - |

Note: Red represents Iden greater than 70 and Sim greater than 80. “-” denotes not tested in this study.
