# Supplementary material for: Comprehensive genome-wide analysis of the pear (Pyrus bretschneideri) laccase gene (PbLAC) family and functional identification of PbLAC1 involved in lignin biosynthesis
Source: PLoS One. 2019 Feb 12;14(2):e0210892. doi: 10.1371/journal.pone.0210892 (PMC6372139; doi:10.1371/journal.pone.0210892)
Supplement: S10 Table — (DOCX) [file pone.0210892.s010.docx]

**Table S10 FPKM values of *PbLAC*s in pear fruit at various developmental stages.**

| **Gene Name** | **Genome ID** | **23 DAF**  **FPKM** | | **55 DAF**  **FPKM** | | **145 DAF**  **FPKM** | |
| --- | --- | --- | --- | --- | --- | --- | --- |
|  |  | **DS** | **LS** | **DS** | **LS** | **DS** | **LS** |
| *PbLAC1* | Pbr003857.1 | 0 | 0.896838 | 88.9576 | 73.3127 | 0.637814 | 0.726981 |
| *PbLAC2* | Pbr010954.1 | 0.155144 | 2.081761 | 12.24453 | 18.23885 | 0.113401 | 0.276382 |
| *PbLAC3* | Pbr010955.1 | 0.154744 | 2.080893 | 12.21609 | 18.27032 | 0.115396 | 0.286093 |
| *PbLAC4* | Pbr011352.1 | 0 | 0.161001 | 9.242913 | 32.24872 | 0.091575 | 0.273167 |
| *PbLAC5* | Pbr042315.1 | 0.19125 | 0.596833 | 63.81047 | 93.51434 | 0.58851 | 0.364619 |
| *PbLAC6* | Pbr012358.1 | 0 | 3.760864 | 327.6365 | 471.0278 | 2.977021 | 2.592634 |
| *PbLAC7* | Pbr012397.1 | 0 | 0 | 0 | 0 | 0 | 0 |
| *PbLAC8* | Pbr012398.1 | 0 | 0 | 0 | 0 | 0 | 0 |
| *PbLAC9* | Pbr013101.1 | 0 | 0.284937 | 0 | 0.122329 | 0.327529 | 0.538357 |
| *PbLAC10* | Pbr013455.1 | 525.8911 | 1330.554 | 11.62174 | 9.536205 | 4.369061 | 3.825213 |
| *PbLAC11* | Pbr013454.1 | 529.266 | 1353.801 | 11.37699 | 9.289386 | 4.291662 | 3.762545 |
| *PbLAC12* | Pbr013456.1 | 0 | 0 | 0 | 0 | 0 | 0 |
| *PbLAC13* | Pbr014327.1 | 0.273467 | 0.061295 | 0.27964 | 0.484081 | 0.129656 | 0.361364 |
| *PbLAC14* | Pbr018935.1 | 0 | 0 | 0.186242 | 0 | 0.104518 | 0.391395 |
| *PbLAC15* | Pbr022396.1 | 0 | 0 | 0 | 0 | 0 | 0 |
| *PbLAC16* | Pbr023439.1 | 0 | 0 | 0 | 0 | 0 | 0 |
| *PbLAC17* | Pbr023440.1 | 0 | 0 | 0 | 0.08754 | 0 | 0 |
| *PbLAC18* | Pbr023443.1 | 0 | 0 | 0.051601 | 0.069398 | 0 | 0 |
| *PbLAC19* | Pbr024489.1 | 4.168085 | 4.213585 | 12.54574 | 13.97092 | 43.13348 | 46.28384 |
| *PbLAC20* | Pbr024725.2 | 48.7205 | 12.3126 | 0.731255 | 2.19928 | 0.1048 | 0.432799 |
| *PbLAC21* | Pbr027989.1 | 0 | 0.030373 | 2.79276 | 5.44716 | 0 | 0.035287 |
| *PbLAC22* | Pbr028392.1 | 0 | 0 | 0 | 0 | 0 | 0 |
| *PbLAC23* | Pbr029302.1 | 0 | 0 | 0.563855 | 0.570252 | 0 | 0 |
| *PbLAC24* | Pbr029309.1 | 0 | 0 | 4.12264 | 5.06834 | 0.034321 | 0.03843 |
| *PbLAC25* | Pbr029312.1 | 0 | 0 | 0 | 0 | 0 | 0 |
| *PbLAC26* | Pbr030875.1 | 9.228143 | 9.457436 | 13.25457 | 11.4683 | 10.90885 | 11.88844 |
| *PbLAC27* | Pbr030877.1 | 0.244791 | 0 | 0.414159 | 0.075889 | 0.034792 | 0 |
| *PbLAC28* | Pbr032053.1 | 0.039889 | 0.420293 | 0.440275 | 0.448827 | 0 | 0 |
| *PbLAC29* | Pbr033951.1 | 0.141203 | 0.869978 | 102.3201 | 98.52771 | 0.931325 | 0.390669 |
| *PbLAC30* | Pbr034913.1 | 0 | 0 | 0 | 0 | 0 | 0 |
| *PbLAC31* | Pbr034948.1 | 0 | 0 | 4.12264 | 5.06834 | 0.034321 | 0.03843 |
| *PbLAC32* | Pbr035745.1 | 0 | 0 | 0 | 0 | 0 | 0 |
| *PbLAC33* | Pbr035748.1 | 0.079473 | 0.150856 | 2.902881 | 6.86282 | 0.052213 | 0 |
| *PbLAC34* | Pbr035749.1 | 0 | 0 | 0 | 0 | 0 | 0 |
| *PbLAC35* | Pbr035929.1 | 0 | 0 | 0 | 0.807124 | 0 | 0.042942 |
| *PbLAC36* | Pbr035962.1 | 0 | 2.436902 | 287.841 | 284.834 | 1.795 | 1.859594 |
| *PbLAC37* | Pbr038866.1 | 0 | 0 | 0 | 0.807308 | 0 | 0.043622 |
| *PbLAC38* | Pbr038988.1 | 0 | 0.39592 | 50.5562 | 54.03716 | 0.361097 | 0.380696 |
| *PbLAC39* | Pbr039980.1 | 437.4065 | 119.5518 | 1.049704 | 1.414235 | 0.678202 | 1.820128 |
| *PbLAC40* | Pbr041372.1 | 0 | 0 | 0 | 0 | 0 | 0 |
| *PbLAC41* | Pbr041924.1 | 0.458547 | 1.206891 | 7.812394 | 19.92579 | 1.109463 | 6.883602 |
