# Supplementary material for: Comprehensive genome-wide analysis of the pear (Pyrus bretschneideri) laccase gene (PbLAC) family and functional identification of PbLAC1 involved in lignin biosynthesis
Source: PLoS One. 2019 Feb 12;14(2):e0210892. doi: 10.1371/journal.pone.0210892 (PMC6372139; doi:10.1371/journal.pone.0210892)
Supplement: S11 Table — (DOCX) [file pone.0210892.s011.docx]

**Table S11 The relevant parameters of each *PbLAC* qRT-PCR primer.**

| **Gene name** | **Amplification efficiency（%）** | **Regression coefficient** | **Standard Curve** | **Melt Curve** |
| --- | --- | --- | --- | --- |
| *PbLAC1* | 110.6 | 0.996 | 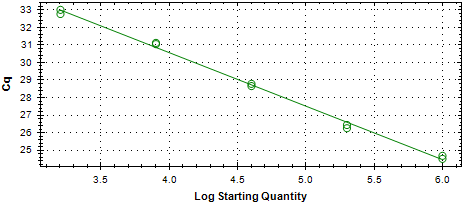 | 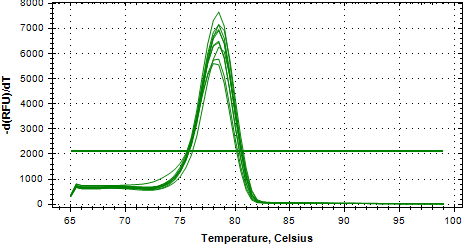 |
| *PbLAC6* | 92.4 | 0.998 | 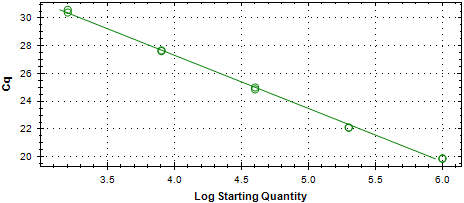 | 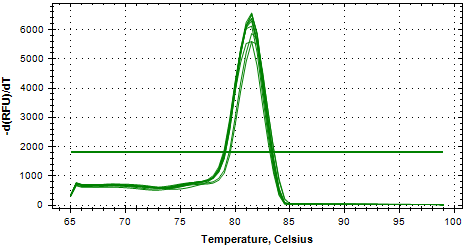 |
| *PbLAC14* | 112.2 | 0.997 | 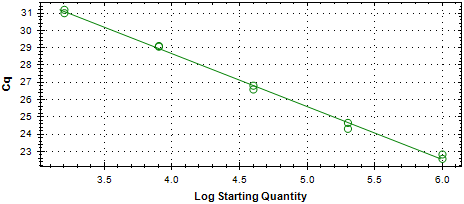 | 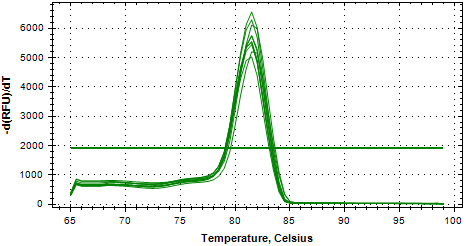 |
| *PbLAC16* | 111.4 | 0.997 | 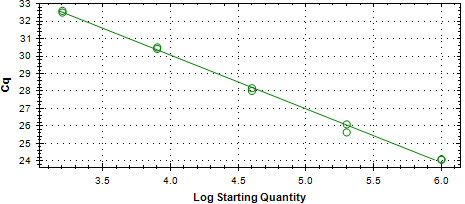 | 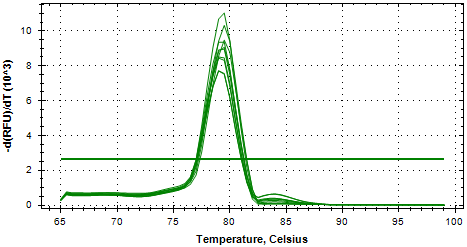 |
| *PbLAC17* | 90.2 | 0.998 | 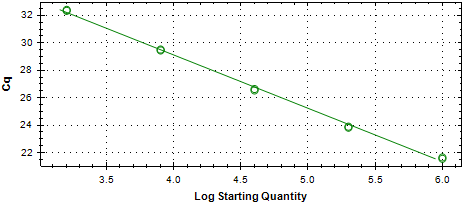 | 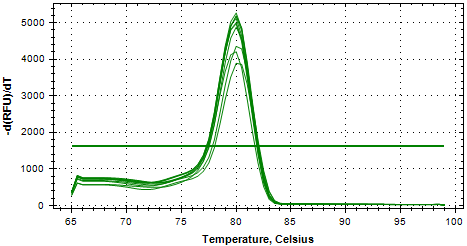 |
| *PbLAC18* | 110.7 | 0.994 | 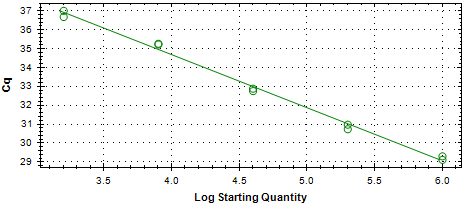 | 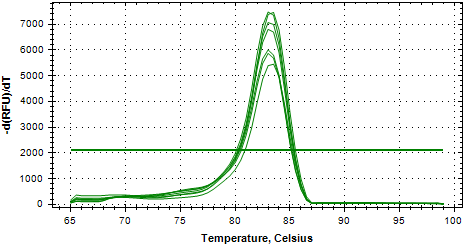 |
| *PbLAC25* | 110.9 | 0.997 | 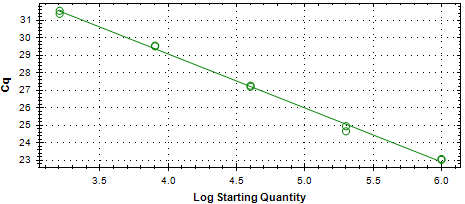 | 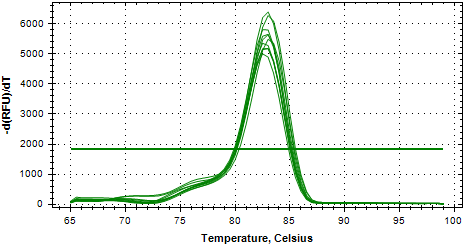 |
| *PbLAC29* | 90.8 | 0.997 | 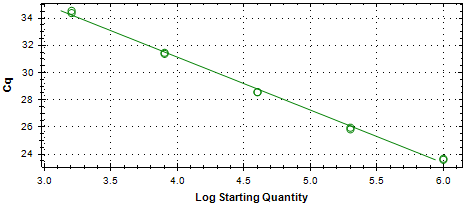 | 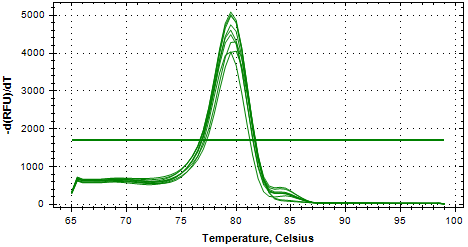 |
| *PbLAC36* | 109.1 | 0.994 | 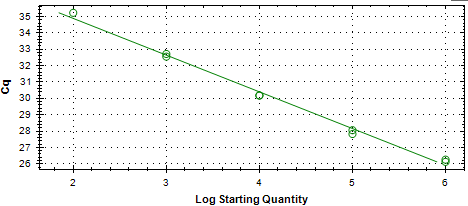 | 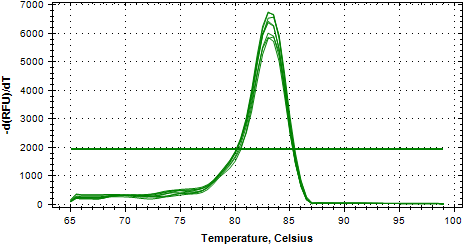 |
