# Supplementary figures and images for: Comprehensive genome-wide analysis of the pear (Pyrus bretschneideri) laccase gene (PbLAC) family and functional identification of PbLAC1 involved in lignin biosynthesis
Source: PLoS One. 2019 Feb 12;14(2):e0210892. doi: 10.1371/journal.pone.0210892 (PMC6372139; doi:10.1371/journal.pone.0210892)

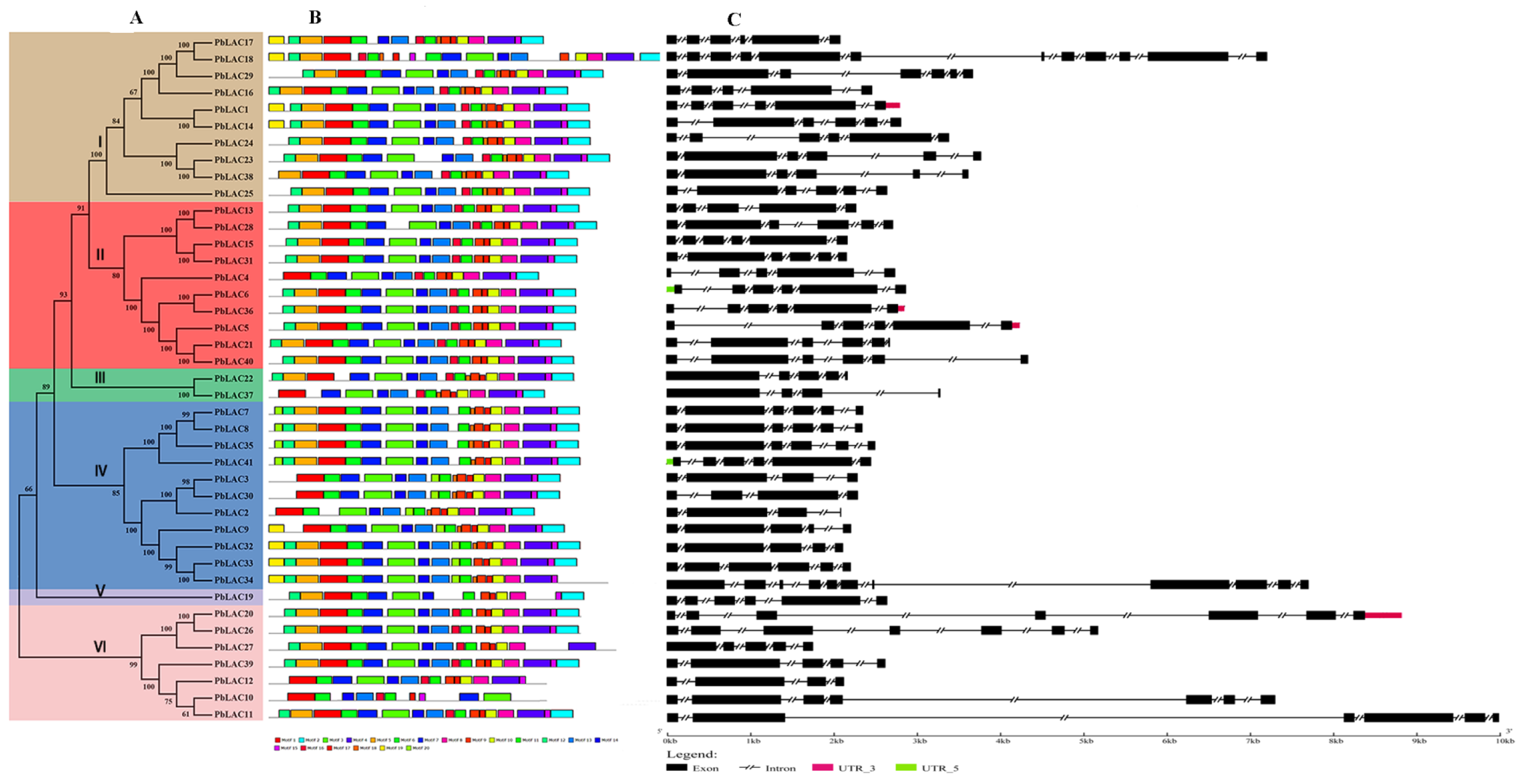

Supplement: S1 Fig — (A) Phylogenetic relationships of PbLACs. (B) Distribution of 20 putative conserved motifs in PbLAC proteins. (C) Exon-intron organization of PbLACs. (TIF) [file pone.0210892.s012.tif]

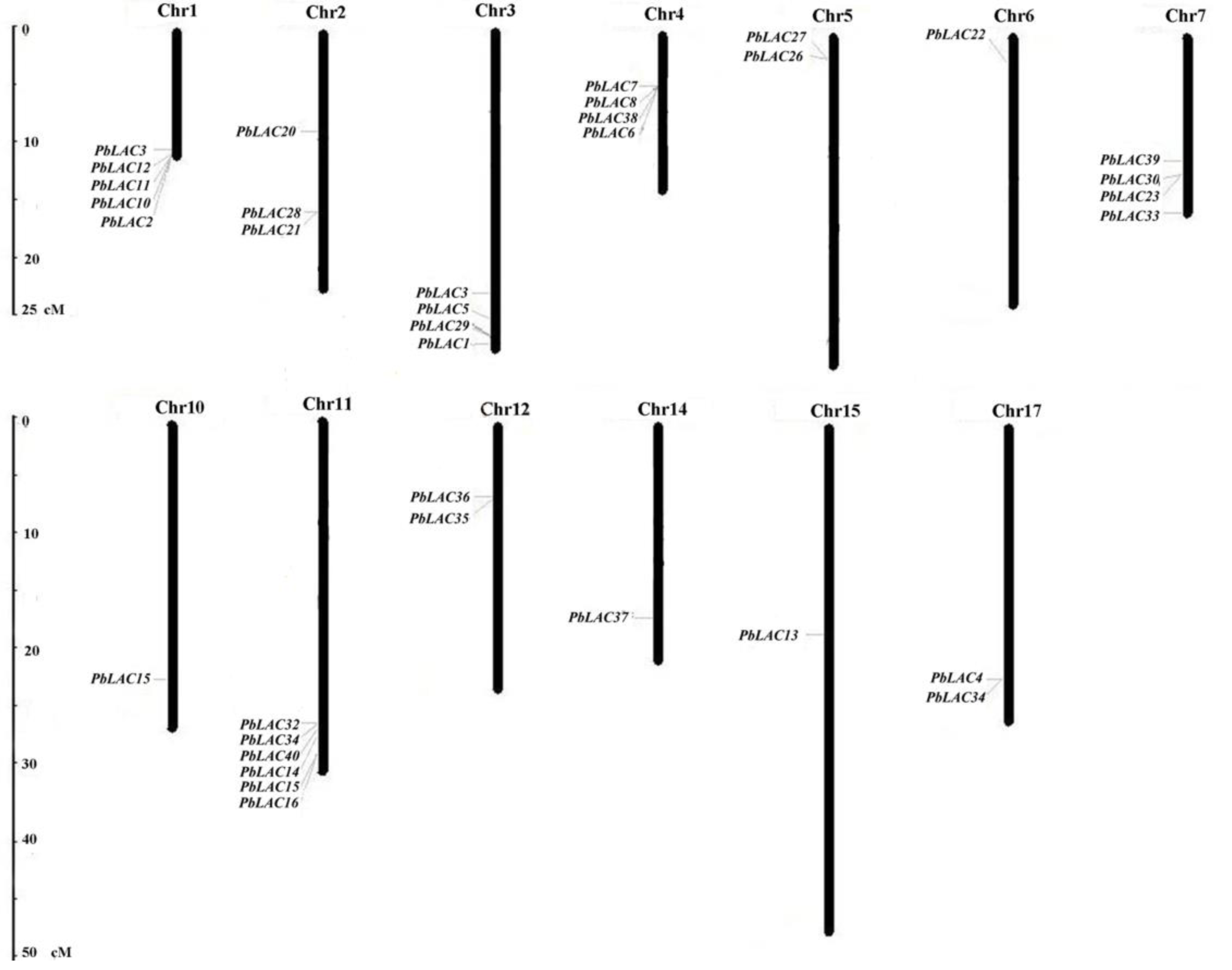

Supplement: S2 Fig — (TIF) [file pone.0210892.s013.tif]

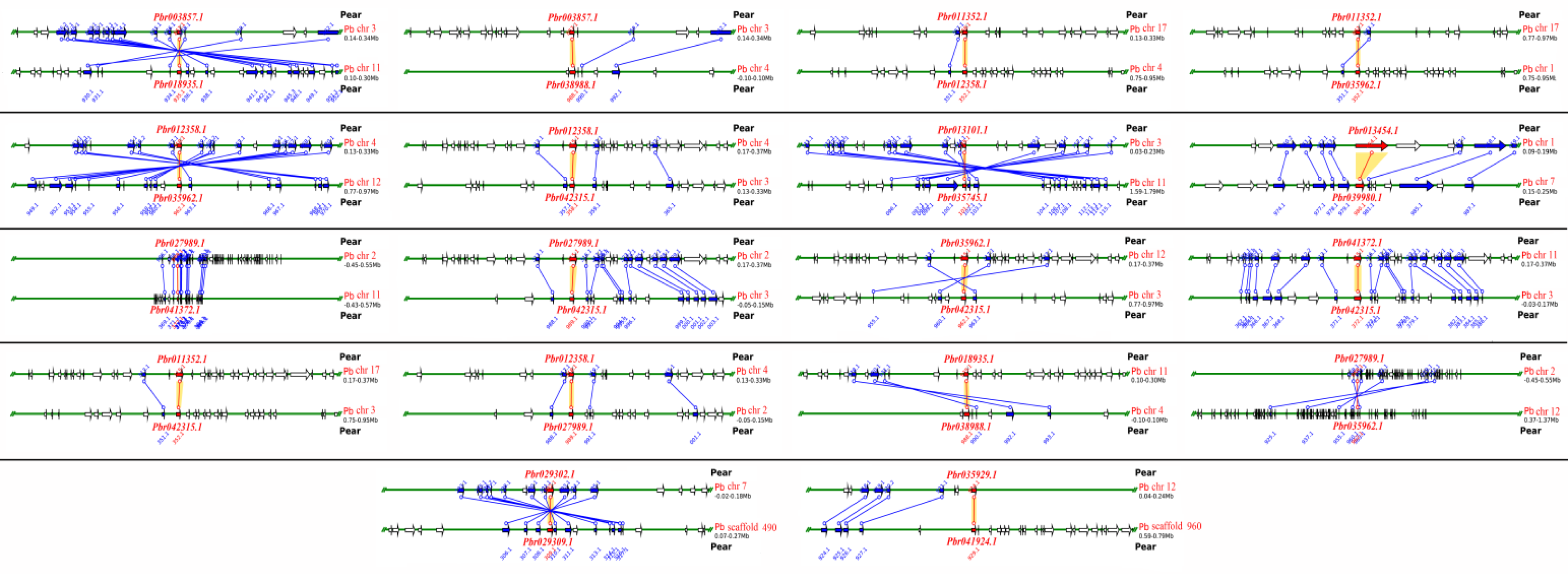

Supplement: S3 Fig — The green bars represent chromosomes, and he chromosome types and regions are shown on the right. The numbers on both sides of the chromosome are the suffixes of the Genome ID of each gene. Homologous gene pairs are connected by straight lines, and the LAC lines are red. The blue lines indicate other anchor genes in the region, and non-homologous genes are shown in white. (TIF) [file pone.0210892.s014.tif]

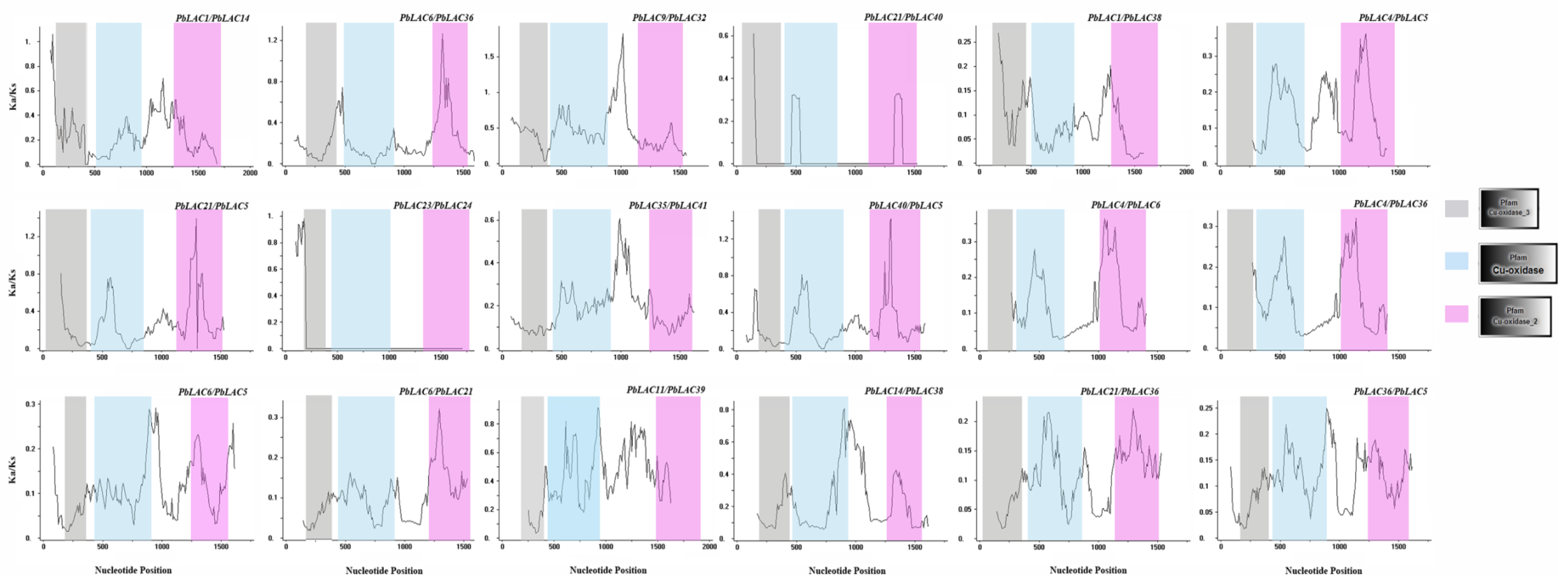

Supplement: S4 Fig — Grey, blue and purple blocks indicate the positions of Cu-oxidase_3 domain, Cu-oxidase domain and Cu-oxidase_2 domain, respectively. The window size was 150 bp, and the step size was 9 bp. The x-axis represents the nucleotide position of the gene pair, and the y-axis represents the Ka/Ks ratio. (TIF) [file pone.0210892.s015.tif]

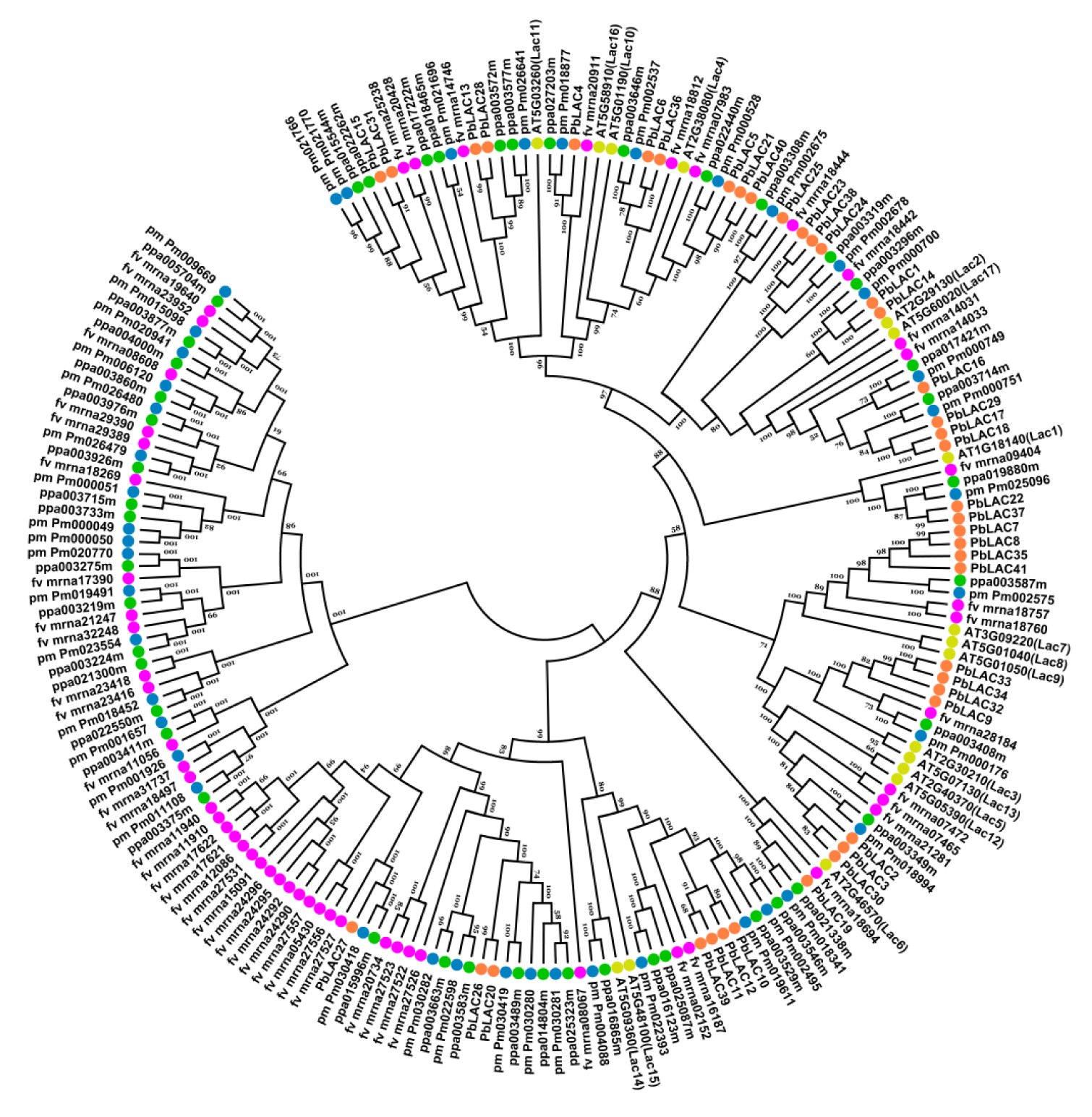

Supplement: S5 Fig — An N-J tree with 41 pear, 54 strawberry, 43 mei, 45 peach and 17 Arabidopsis LAC proteins was created using MEGA5.0. (TIF) [file pone.0210892.s016.tif]

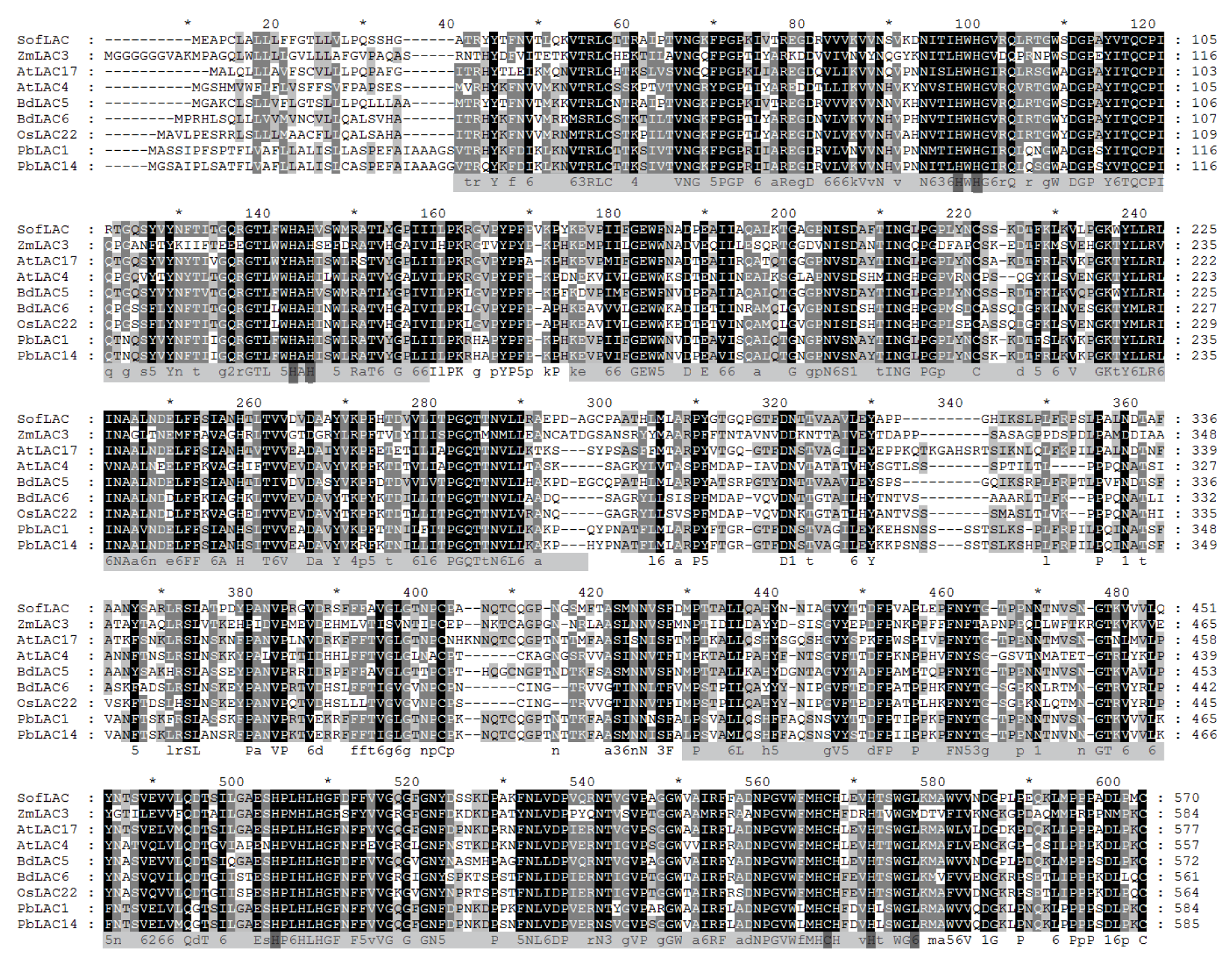

Supplement: S6 Fig — The three consecutive grey-shaded areas are the three conserved domains, namely, CuRO_1_LCC_Plant, CuRO_2_LCC_Plant and CuRO_3_LCC_Plant. (TIF) [file pone.0210892.s017.tif]

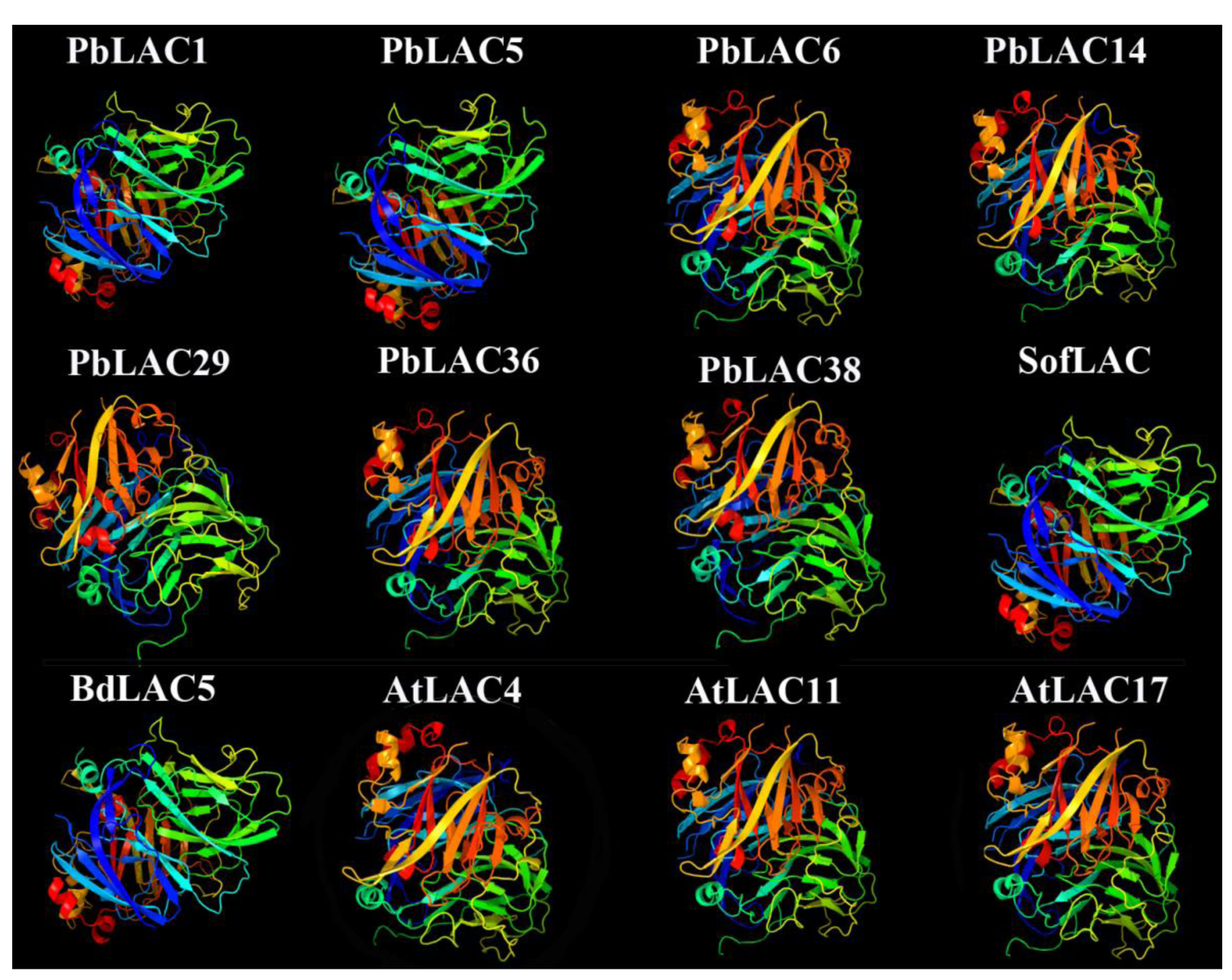

Supplement: S7 Fig — AtLAC4, AtLAC11, AtLAC 17, BdLAC5 and SofLAC have been proven to be responsible for lignin biosynthesis. (TIF) [file pone.0210892.s018.tif]
